# Electrocatalytic activity of metal encapsulated, doped, and engineered fullerene-based nanostructured materials towards hydrogen evolution reaction

**DOI:** 10.1038/s41598-022-20048-3

**Published:** 2022-09-16

**Authors:** Hitler Louis, Onyinye J. Ikenyirimba, Tomsmith O. Unimuke, Gideon E. Mathias, Terkumbur E. Gber, Adedapo S. Adeyinka

**Affiliations:** 1 Computational and Bio-Simulation Research Group, University of Calabar, Calabar, Nigeria; 2 Department of Pure and Applied Chemistry, University of Calabar, Calabar, Nigeria; 3 Research Centre for Synthesis and Catalysis, Department of Chemical Sciences, University of Johannesburg, Johannesburg, South Africa

**Keywords:** Chemistry, Energy science and technology

## Abstract

The utilization of nanostructured materials as efficient catalyst for several processes has increased tremendously, and carbon-based nanostructured materials encompassing fullerene and its derivatives have been observed to possess enhanced catalytic activity when engineered with doping or decorated with metals, thus making them one of the most promising nanocage catalyst for hydrogen evolution reaction (HER) during electro-catalysis. Prompted by these, and the reported electrochemical, electronic and stability advantage, an attempt is put forward herein to inspect the metal encapsulated, doped, and decorated dependent HER activity of C_24_ engineered nanostructured materials as effective electro-catalyst for HER. Density functional theory (DFT) calculations have been utilized to evaluate the catalytic hydrogen evolution reaction activity of four proposed bare systems: fullerene (C_24_), calcium encapsulated fullerene (Ca^enc^C_24_), nickel-doped calcium encapsulated fullerene (Ni^dop^Ca^enc^C_24_), and silver decorated nickel-doped calcium encapsulated (Ag^dec^Ni^dop^Ca^enc^C_24_) engineered nanostructured materials at the TPSSh/GenECP/6-311+G(d,p)/LanL2DZ level of theory. The obtained results divulged that, a potential decrease in energy gap (E_gap_) occurred in the bare systems, while a sparing increase was observed upon adsorption of hydrogen onto the surfaces, these surfaces where also observed to maintain the least E_H–L_ gap while the Ag^dec^Ni^dop^Ca^enc^C_24_ surface exhibited an increased electrocatalytic activity when compared to others. The results also showed that the electronic properties of the systems evinced a correspondent result with their electrochemical properties, the Ag-decorated surface also exhibited a proficient adsorption energy $({E}_{ads}^{H})$ and Gibb’s free energy (ΔG_H_) value. The engineered Ag-decorated and Ni-doped systems were found to possess both good surface stability and excellent electro-catalytic property for HER activities.

## Introduction

In recent years, fullerene which is a carbon-based material has emerged as a potential candidate for use as noble-metal free electrocatalysts for the hydrogen evolution reaction (HER, 2H + (aq) + 2e ⇄ H2(g)), which is a cathodic half-reaction of water splitting^1,2^. This is due to the enormous importance of fullerene which include low price, abundant reserves, and long- term stability^3^. Hydrogen has been known as a key energy carrier, and its electrocatalytic reaction is a cathodic reaction in water electrolysis, while its anodic reaction which is hydrogen oxidation reaction (HOR) is an anodic reaction in fuel cells^4^. Through research, it has recently been found that hydrogen is becoming a significant competitor as a stable and non-carbon energy source in the renewable energy grid^5,6^.

Density functional theory (DFT) have recently been used by many scientists in parallel to the development of experimental strategies, this is due to the facts that theoretical calculation takes into account the surface (electro) chemistry and electronic structure and materials that have been essential for improving the fundamental understanding of HER electrocatalysis. Pier Paolo et al.^7^ performed Electrochemical studies of hydrogen evolution, storage and oxidation on carbon nanotube electrodes and reported that hydrogen is easily produced on the carbon nanotube surface, but a significant overvoltage according to them was observed for hydrogen oxidation. DFT studies on Mechanisms of fullerene and single-walled carbon nanotube composite as the metal-free multifunctional electrocatalyst for the oxygen reduction, oxygen evolution, and hydrogen evolution were conducted by Xin Chena et al.^8^ and their obtained results showed that hydrogen adsorption free energy was calculated to be a small value of 0.39 eV, also indicating the high hydrogen evolution activity of the site-2. Advancing the Electrochemistry of the Hydrogen Evolution Reaction (HER) through Combining Experiment and Theory were conducted by Yao Zheng et al.^9^ and their result shows that DFT computations were powerful in predicting the capability of these materials, paving the way toward molecular design of catalysts for HER. Similarly, DFT approach was employed by Alain R. Puente^10^ in the study of A New Class of Molecular Electrocatalysts for Hydrogen Evolution: Catalytic Activity of M_3N_@C_2n_ (2n = 68, 78, and 80) Fullerenes and determined that the non-IPR Sc_3N_@ D3(6140)-C_68_ exhibits an impressive HER activity. Using periodic Density Functional theory by employing the new Bayesian error estimation functional with van der Waals correlation (BEEF-vdW) functional, Charlie Tsai et al.^11^ evaluated the tuning of MoS_2_ Edge-Site activity for Hydrogen Evolution via Support Interactions and found that the support interactions involving vdW forces leads to significant changes in the hydrogen binding energy, resulting in several orders of magnitude difference in HER activity. Jun Xing et al.^5^ studied the active sites on hydrogen evolution photocatalyst and reported through a combined experimental approach, that metallic Pt nanoparticles have little contribution to the activity of photocatalytic H_2_ evolution; the oxidized Pt species embedded on the TiO_2_ surface are the key active sites and primarily responsible for the activity of the hydrogen evolution Pt/TiO_2_ photocatalyst. DFT approach was also employed by Puente et al.^12,13^ in the scientific area of “Tailoring the interfacial interactions of van der Waals 1T-MoS_2_/C_60_ Heterostructures for High-Performance Hydrogen Evolution Reaction Electrocatalysis and their result showed that the heterostructure domains of 1 T-MoS_2_ and C_60_ NSs exhibited excellent hydrogen evolution reaction (HER) performances, with one of the lowest onset potentials and ΔGH* values for LD non-precious nanomaterials. In furtherance, Lin et al., proposed alloying noble and non-noble metals as promising catalyst for HER. The enhanced HER activity was realized by the fabrication of Ir1-xRhxSb alloy centres via the inducement of negatively charged centres which resulted in remarkable electron transfer and considerable adsorption of active H species during HER as a consequence, Tafel slope of about 47.6 mV dec^−1^ and overpotential of 22 mV at 10 mA cm^−2^ were achieved. In similar manner, the authors utilized a one-step approach with an ultrafast pulsed laser treatment to enhance the HER activity by multiple decorations of lamellar MoS_2_. As a result of micro structure and modification, the catalytic activity of the laser-treated MoS_2_ was found to be superior when compared to the untreated lamellar MoS_2_^14,15^.

Despite this remarkable and extraordinary progress in density functional theory (DFT) results, the electrocatalytic performance of the engineered fullerene nanomaterials for electrochemical hydrogen evolution (H@Ag^dec^Ni^dop^Ca^en^C_24_) has not been theoretically elucidated. Therefore, we have considerably studied the electrocatalytic HER properties of fullerene engineered nanomaterials in this present study to have a clear insight into their electronic, physical, and electrochemical properties. Nowadays, understanding the electronic characteristics and the intermolecular interaction in nanomaterials is essential for both theoretical researchers and experimentalists, hence in this work we have attempted to evaluate the electronic properties of the fullerene engineered nanomaterials via the Frontier Molecular Orbital analysis (FMO), the donor–acceptor interaction between the fullerene and catalytic Hydrogen have been studied using the natural bond orbital analysis (NBO), electronic distribution and the electron density distribution by Density of state (DOS) and Quantum theory of atoms in molecules (QTAIM). The computer-aided design of catalysts through DFT has been utilized herein to study some theoretical concepts such as adsorption energy(s) calculation, from which a detailed understanding of the adsorption energy of hydrogen on the engineered fullerene surfaces has been here by calculating the adsorption energy of the atomic hydrogen, molecular hydrogen and the dissociative hydrogen, Gibb’s free energy, energy barrier, surface model and transition state to quantitatively evaluate the performance of Hydrogen Evolution Reaction electro-catalysts.

## Computational details

All computational calculations performed in this study were estimated within the framework of Density functional theory implemented with the help of Gaussian 16 program^16^. Ground state geometry optimization was carried out using TPSSh, the exchange functional of Tao, Perdew, Staroverov, and Scuseria which is a Meta-generalized gradient approximation (M-GGA) functional exchange^17^, TPSSh has been reported to be good for geometry optimization and posess good accuracy score on the Jacobi ladder of DFT functionals hence; the choice of this functional in this study. Transition state calculation was performed on the same model using the optimization and frequency job type optimize to TS(QST2). All the system studied here were calculated in vacuum using the GenECP methods by assigning the 6-311++G(d,p) and the LanL2DZ basis set for the lighter and heavier atoms respectively^18^. A deep understanding of the electronic characteristic of the electrochemical hydrogen evolution (H@Ag^dec^Ni^dop^Ca^en^C_24_) was studied by employing the frontier molecular orbital analysis. The changes in the electronic energy and the orbital energies have been provided using the GaussSum 3.0 package^19^ from the plots, a clear distinct difference between the virtual and the occupied molecular region was observed. To gain a deeper insight into the nature of the inter-atomic interactions, the topological analysis—Quantum Theory of Atoms in Molecules (QTAIM) analyses and DOS plots which gave much enlightenment on the precise state where electrons can be located were completed with the help of Multiwfn 3.7 program^20^. Meanwhile, pictorial representations of frontier molecular orbital analysis from electronic studies were visualized using the visual molecular dynamics (VMD) software^21^. To explore the knowledge of the donor–acceptor interactions, (NBO) analysis were calculated using NBO 7.0 package embedded in Gaussian 16.^22^ Geometry structure analysis was visualized using chemcraft 1.6 package. The electrochemical calculation including Gibb’s free energy, Surface model, Energy barrier were computed at the same level of theory. The adsorption energy (E_ad_) for the complexes from the interactions of catalytic hydrogen with the engineered fullerene nanomaterials including the atomic hydrogen, molecular hydrogen and the dissociative hydrogen states were computed using the equation:

1 $$E_{ads}^{H} = {\text{ E}}_{{({\text{Total}})}} - {\text{ E}}_{{({\text{Surface}})}} {-}\frac{1}{2}{\text{E}}_{{({\text{H2}})}}$$

## Results and discussion

### Geometry and structural analysis of the studied C_24_ and engineered C_24_ surface

**Figure 1:**
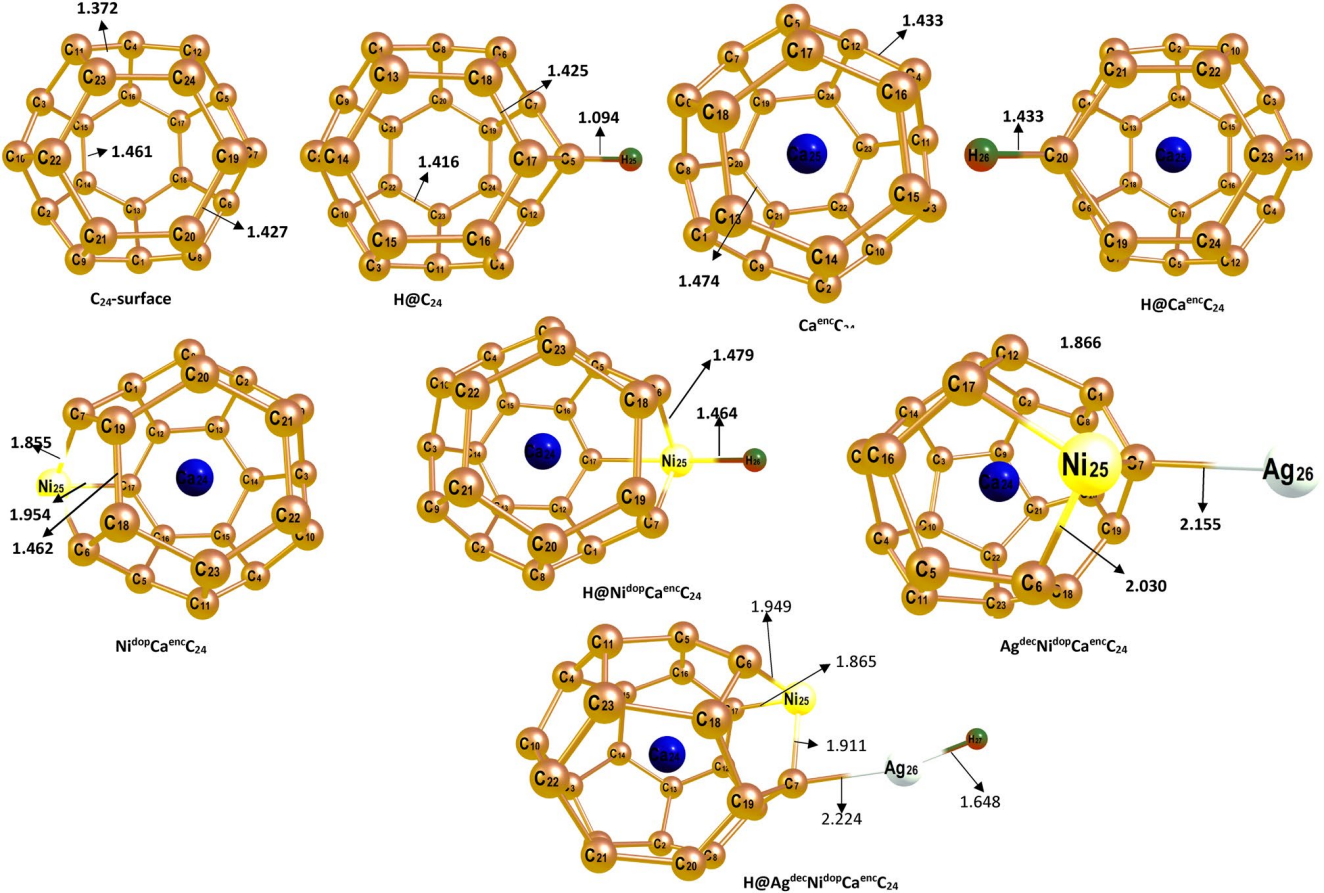
Optimized geometry of the bare C_24_ surface, and engineered C_24_ surfaces.

The configuration of four (4) systems (C_24_, C_24_-Engineered-Ca-encapsulated, Ni-doped, and Ag-decorated) surfaces were optimized to their preferable and stable geometry before going on to the H-adsorption processes and was evaluated using the DFT/TPPSh /GenECP level. The titled surface of interest; C_24_ is a hollow structure embracing a hexagonal ring structure with surrounding carbon atoms in its entirety. Hence, consisting of interfacial C–C bonds, with bond distances of 1.427 Ấ for the innermost bonds, and 1.461 Ấ for the outer bond, ranging from one carbon to the next between the hexagonal ring. In furtherance, the encapsulated, doping, and decoration of the C_24_ surface, which was piloted at different stable positions initiated into the C_24_ surface for the encapsulated system, replacing one carbon atom without causing defects to the fullerene surface, while the doped Ni atom was shot at a very stable configuration into the C_24_ surface taking atom number 25, forming a bond angle of 2.030 Ấ on the pentagonal ring with linked carbon atoms of the C_24_ system, and lastly, the decorated Ag atom without distortion formed C–Ag bond length with C_7_ of the Ca^enc^C_24_ system, which is of 2.155 Ấ. Thus, the geometrical position of the encapsulated, doped, and decorated Ca, Ni, and Ag atoms are basically inside and by the side of a pentagonal ring in the fullerene system. As such, the various engineered atoms after geometrical optimization at the various preferable positions results depicted that the adsorbed engineered metal of interest; Ca, Ni, and Ag preferably was adsorbed on the carbon atoms as a result of the high electronegative nature of carbon atoms, with an electronegative value of 2.55 according to Paulings. Thus, giving access to the highly electropositive studied Ca, Ni, and Ag-engineered metals of interest to bind conjugatively within the surface, causing an elongation in bond length, which lucidly explains the phenomenon of charge transfer from the adsorbed engineered metal-atoms to the fullerene surface, which is attested to by the charge transfer from the adsorbate to the adsorbent, bearing a negative (-) charge as also reported in the literature^2^. Interestingly, for the titled Ni^dop^Ca^enc^C_24_, and Ag^dec^Ni^dop^Ca^enc^C_24_, the bond between Ni_25_–C_17_ and Ag–C are 2.030 Ấ and 2.155 Ấ respectively, with corresponding NBO electron transfers of − 0.012é, − 0.0045é, and − 0.1100é respectively. As such, a representative illustration of the geometry of the studied surfaces with their atomic labeling, and selected bond lengths are shown in Fig. 1. Also, each surface was observed to be stabilized by zero chare and multiplicity of 1e except for the Ag-decorated surface which is stabilized by a spin multiplicity of 2. All the engineered surfaces were equally observed to be of C1 symmetry while the C_24_ surface maintained a D2 symmetry throughout the optimization process.

### Electronic properties

#### Reactivity analysis

**Figure 2:**
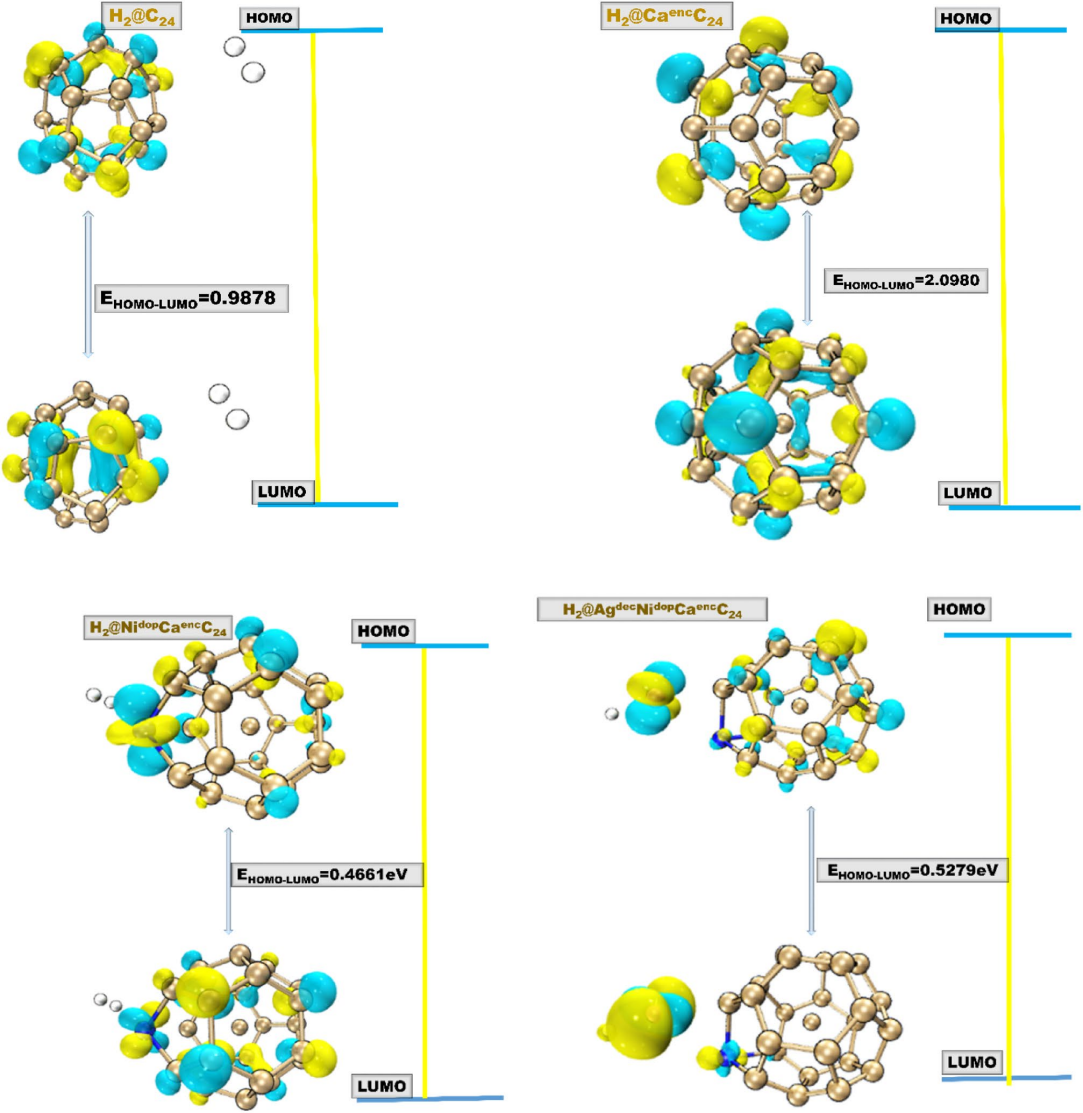
HOMO–LUMO distribution of the studied hydrogen adsorbed systems, with their significant HOMO–LUMO energy gap (E_gap_).

Analysis of global quantum molecular descriptors^23–25^ was officiated for this study to gain additional details related to adsorption of hydrogen by the S_1_-S_4_ surface, and the hydrogenated systems. The study laid emphasis on HOMO energies, LUMO energies, HOMO–LUMO energy gap (E_g_), and the electrophilicity index. Thus, in order to obtain the electrophilicity index, the calculation of chemical potential (μ) was computed. Hence, energy gap representing the maximum energy difference between HOMO and LUMO, electrophilicity index was calculated in line with previous reports based on Koopman’s hypothesis. Based on Koopmans theorem, the HOMO and LUMO energies gave lucid information about the stability and activity of the studied systems. HOMO represents the tendency to donate an electron, while the LUMO acts as an electron acceptor, instigating the ability to accept electrons. The changes in the electronic conductivity of S_1_, S_2_, S_3_, and S_4_, in conjunction with H_c_-H_Ag*_ were studied by FMO analysis, providing distinctive information about the electrocatalytic hydrogen evolution reaction abilities of the studied systems. The HOMO–LUMO iso-surface plot of the systems is given in Fig. 2, and their reactivity energies are presented in Table 1. The reactivity energy values of the HOMO and LUMO of the bare surfaces and the hydrogenated surfaces are given in Table 1 in the succeeding sections (“HOMO–LUMO energy gap study” section), representatively. As such, having energy gap values of 0.5549, 1.9650, 0.5628, 0.4882 eV for the bare surfaces studied, and 0.9878, 2.0980, 0.5279, and 0.4661 eV for the hydrogenated systems.

**Table 1:** The energies of the HOMO (eV), LUMO (eV), energy gap (E_g_) and electronic NBO stabilization energies of the studied systems.

Systems	E_HOMO_ (eV)	E_LUMO_ (eV)	E_g_ (eV)	µ	ω	ΣNBO (E^2^)
C_24_ (S_1_)	− 5.3713	− 4.8164	0.5549	− 5.0939	6.0888	ΣE^(2)^ = 43.46
Ca^enc^C_24_ (S_2_)	− 6.2143	− 4.2493	1.9650	− 5.2318	11.9820	ΣE^(2)^ = 177.00
Ni^do^Ca^enc^C_24_ (S_3_)	− 5.2072	− 4.6444	0.5628	− 4.9259	5.9449	ΣE^(2)^ = 269.64
Ag^dec^Ni^do^Ca^enc^C_24_(S_4_)	− 4.6649	− 4.1767	0.4882	− 4.4208	5.2983	ΣE^(2)^ = 155.00
H@Ca^en^C_24_ (H_C_)	− 5.1283	− 4.1405	0.9878	− 4.6344	6.8123	ΣE^(2)^ = 169.80
H@Ca^en^C_24_ (H_Ca*_)	− 6.1914	− 4.0934	2.0980	− 5.1424	13.2526	ΣE^(2)^ = 153.40
H@Ni^dop^Ca^en^C_24_ (H_Ni*_)	− 5.0866	− 4.6205	0.4661	− 4.8536	5.6705	ΣE^(2)^ = 158.79
H@Ag^dec^Ni^dop^Ca^en^C_24_(H_Ag*_)	− 5.5015	− 4.9737	0.5279	− 5.2377	6.1707	ΣE^(2)^ = 220.63

**Figure 3:**
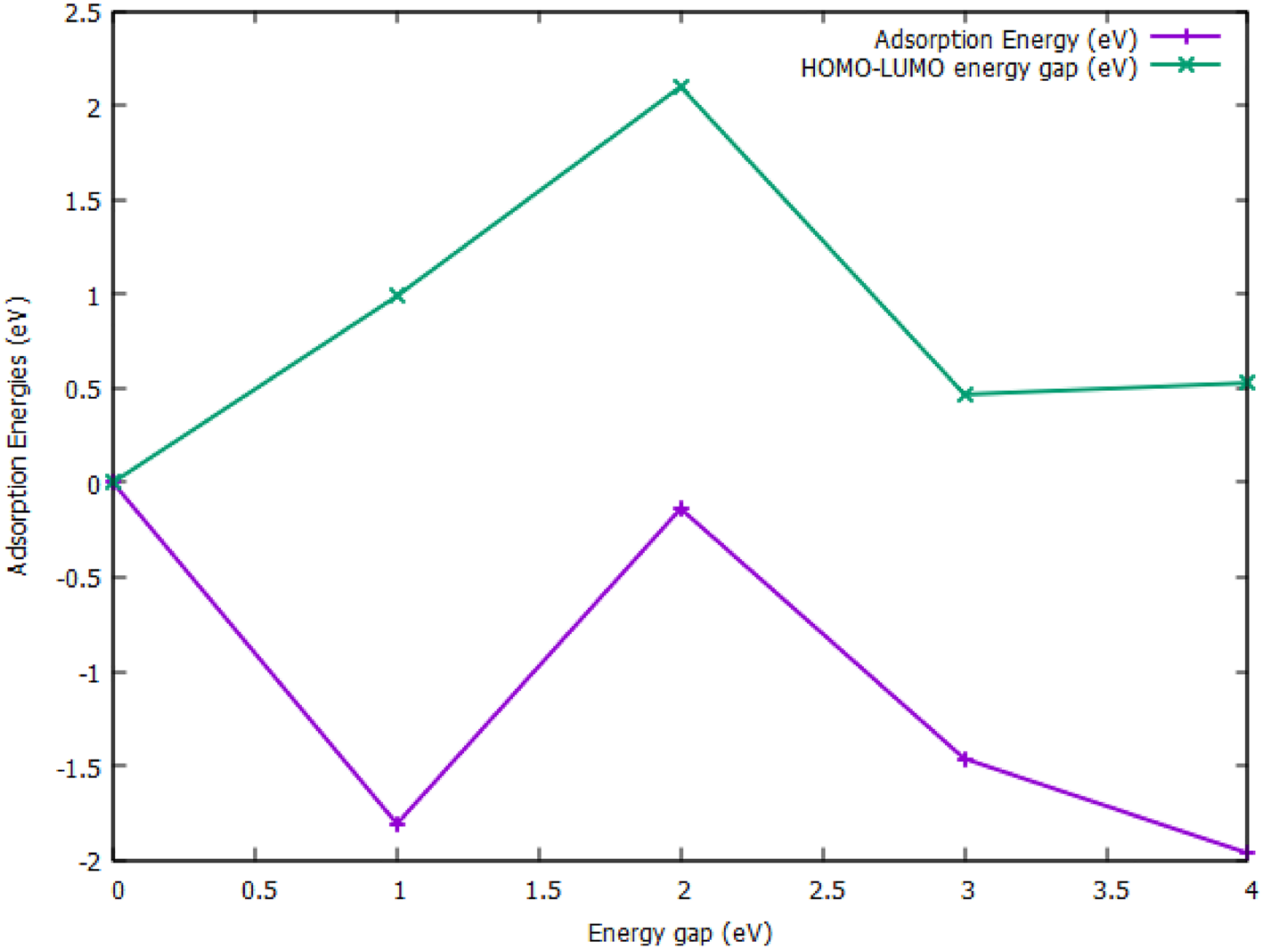
Graph of adsorption energy cum HOMO–LUMO energy gap of the studied engineered Fullerene- system showing the relationship between ${E}_{ads}^{H}$ and E_HOMO–LUMO_.

In furtherance, the adsorption of hydrogen on the titled surfaces increases the HOMO–LUMO energy gaps to the values depicted in Table 1. Hence, the proportionate decrease in the HOMO, and LUMO energy values of the studied surfaces before adsorption resulted in a significant change in energy gaps, thus a pronounced increase was observed for the decorated Ag-surface after H-adsorption. As such, the noticeable decrease in the LUMO energy of the Ag^dec^ bare surface before adsorption and its increase after adsorption is relegated to the major shift observed in the energy gap (E_gap_) of Ag^dec.^ Ni^dop^C^enc^C towards hydrogen evolution reaction. The reactivity electronic distribution analogy of the studied systems obtained through reactivity analysis is given in Fig. 3. The HOMO density is entirely located on the engineered surfaces, while the LUMO iso-surface mapping is present on the engineered and surrounding C-atoms. This illustrated a significant increase in the energy gap of the studied systems, showing that a potential increase is observed when the electronic transition occurred from the surface to the hydrogenated systems. Hence, literature has shown that this kind of energy shift is a great criterion for proficient electrocatalytic activity.

#### Insight into the Highest occupied molecular orbital (HOMO)

The transition strength of electrons from the topmost of the valence band to the bottom of the conduction band and electronic structure through HOMO and LUMO was investigated. To elucidate the nature of the difference between hydrogen adsorption on the studied surfaces and their interactions with atomic hydrogen, and their adsorption sites. Reactivity analyses were carried out on the hollow Ca^enc^, Ni^dop^, Ag^dec^, and the hydrogen atom. The result thus, indicated that the HOMO energies for the studied surfaces as depicted in Table 1 are relatively in correspondence with slight negativity differences. The HOMO energies of the S_1_–S_4_ were theoretically calculated to have the reactivity values of − 5.3713, −6.2143, −5.2072, and −4.6649 eV respectively. The lowest value of HOMO was observed for the decorated Ag surface. Hence, yielding the least HOMO–LUMO energy gap (0.4882 eV), also the tentative result obtained indicated a slight decrease in the value of the HOMO energies when adsorbed with atomic hydrogen in the order H_ca*_ < H_Ni*_ < H_C_ > H_Ag*_. As such, an exceptional energy value was observed for the H_Ag*_ surface, with a less negative value. These values indicate that the HOMO energies sparsely decrease, and the iso-surface mapping of the molecular orbital densely located on the engineered encapsulated, doped, and decorated atoms as shown in Fig. 3. More so, as illustrated in the reactivity table, the decrement in the highest occupied molecular orbital energy before and the meagre increase after H adsorption can be attributed to the non-rigid occupancy of the occupied π- electrons in large conjugated π-orbital systems, bringing about the distribution of energy throughout the molecule. Thus, stabilizing the systems, and also due to the adsorption process. Moreover, the increase in negativity of the HOMO energy of the decorated Ag surface can be attributed to the reactivity oxidation state of Ag in relation to the other doped and encapsulated surfaces, hence as Ag normally exists in the + 1 state will hence tend to be more reactive than the other engineered surfaces. As such increasing the negativity of the HOMO energy of the surface in contact with the adsorption of atomic hydrogen.

The distribution of the HOMO mapping obviously shows an equal distribution on the engineered atoms of interest in both the titled surfaces and the H-adsorbed systems, with an indication that the sites are active to interact with the HOMO of the H-atom, with regards to the surfaces before adsorption of a hydrogen atom, and also indicating chemisorption, and the hydrogen atom acting as an electron acceptor. It is also, worthy to emphasize that the Ag-surface exhibited the least HOMO energy value and exhibited very strong adsorption energy, manifesting its relative interaction with the adsorbed hydrogen atom.

#### Lowest unoccupied molecular orbital (LUMO)

The impact of the quantum chemical calculation protocol was assessed with a subset of the studied surfaces and engineered titled encapsulated, doped, and decorated Ca, Ni, and Ag surfaces. Inclusion of the LUMO orbital energies calculated at split 6-311++G(d,p)/Gen (Auto) theoretical level was investigated. The S_1_–S_4_ titled surfaces in Table 1 showed a relative and correspondence LUMO energy in the trend −4.8164, −4.2493, −4.6444, and −4.1767 eV for the studied surfaces, sprouting an increase in LUMO energy which is in tandem to the to higher negativity value of the LUMO energies. Hence, the increment in LUMO energy after adsorption of the atomic hydrogen indicates a variable transition of the bonding orbitals to the anti-bonding orbital, and the adsorption process also contributes to the observed changes. Also, a great increase was observed in the LUMO energy of the Ag decorated surface from having a LUMO energy of −4.1767 to an increased negative energy value of −4.9737 eV when adsorption of atomic hydrogen was infused. The disparity in the HOMO energy of the studied systems and their LUMO energies indicates that more energy is required for the transition of the S_1_–S_3_ surface to an unoccupied molecular orbital than the Ag surface, having lower HOMO energy and a higher LUMO energy, indicating the more possibility of electron transition and the reduction of electrical resistivity of the systems during the adsorption process. These phenomena of the LUMO energy value can be traceable to the adsorption capacities of the studied systems, hence H_Ag*_ having the more adsorption ability can be relegated to its lowest un-occupying molecular orbital acceptance of electron. With respect to the LUMO iso-surface mapping, the LUMO distribution for the studied surfaces and systems was also found to be concentrated on the engineered surfaces (the encapsulated, doped, and decorated atoms) and on the carbon (C) atoms of the titled surfaces. Thus, the H_Ag*_ showed a distinct interaction with the atomic hydrogen, a kind of water splitting. Considerable adsorption energies before, and after adsorption also portrayed the relationship of the LUMO reactivity parameter on the studied surfaces before and after adsorption. As such, the adsorption energy in the order where H_Ag*_ (decorated Ag) shows the least adsorption energy value and maximum adsorption impact, reflects in its LUMO descriptor parameter having the least LUMO energy value. Hence, indicating a fluent relation between adsorption and HER activity.

#### HOMO–LUMO energy gap study

The study also sought to elucidate the correlation between adsorption energies of the studied systems and their respective HOMO–LUMO band gaps before and after adsorption onto hydrogen. The HOMO–LUMO energy gap (Eg) which have a significant influence on the chemical reactivity of the systems. It thus measures the extent of resistance to the change in the electron distribution of the studied systems^26–28^. As shown in Table 1, a comparative study of the calculated HOMO–LUMO energy gap (Eg) of both bare and H-adsorbed systems was studied. The Eg showed a relationship between the adsorption energies and the energy gap of the studied engineered surfaces and systems. As a general transition, HOMO–LUMO energy increases with the adsorption of hydrogen to their hollow surfaces. The system which shows the least adsorption energy and high adsorption capacity; which is the Ag-decorated surface exhibited the least HOMO–LUMO energy gap of 0.4882, and 0.4661 eV, respectively. The system exhibited a great relationship between their adsorption energies and their E_g_ as shown in the absorption energy-Eg graph in Fig. 3. The most fascinating and interesting fact is that the systems that are named good candidates for catalytic hydrogen evolution reaction are similar to or even greater than their hydrogen adsorbed counterparts (E_g_ $\le$ 0.1, indicating the intact electron distribution of the systems under study, upon H-adsorption). HOMO and LUMO energies increased after absorbing the adsorbent (hydrogen) with a slight increase in their energy gap. The energy gap showed an obvious change in the engineered surfaces, owing to the fact that the adsorption process increases the energy band gap values, illustrating a more stable adsorption ability of the studied systems and leading to electron enrichment of the adsorbed H-systems, and the Eg of the titled systems after adsorption showed a visible increment in the energy band gap of H_C_, H_Ca*_, and H_Ni*_, limiting the performance of S_1_, S_2_, and S_4_ surfaces for HER. Hence, attributing more hydrogen evolution reaction activity to the engineered surfaces before adsorption, owing to the fact that their band-gap energies are low and indicating a more stable system for electrocatalytic activity, with the exception of the S_3_ surface, which exhibited a low energy gap before adsorption, and slight E_g_ decrease after H-adsorption. The increment in the width of energy gap increasing to 0.9878, 2.0980, 0. for the H-adsorbed H_a_, H_b_, and H_d_ surfaces indicates that the adsorption energies of H-molecules increased the titled systems conductivities to a minimal extent. With respect to this, all adsorption results of hydrogen are consistent with the conclusion of the corresponding adsorption energies, and other reactivity parameters.

**Figure 4:**
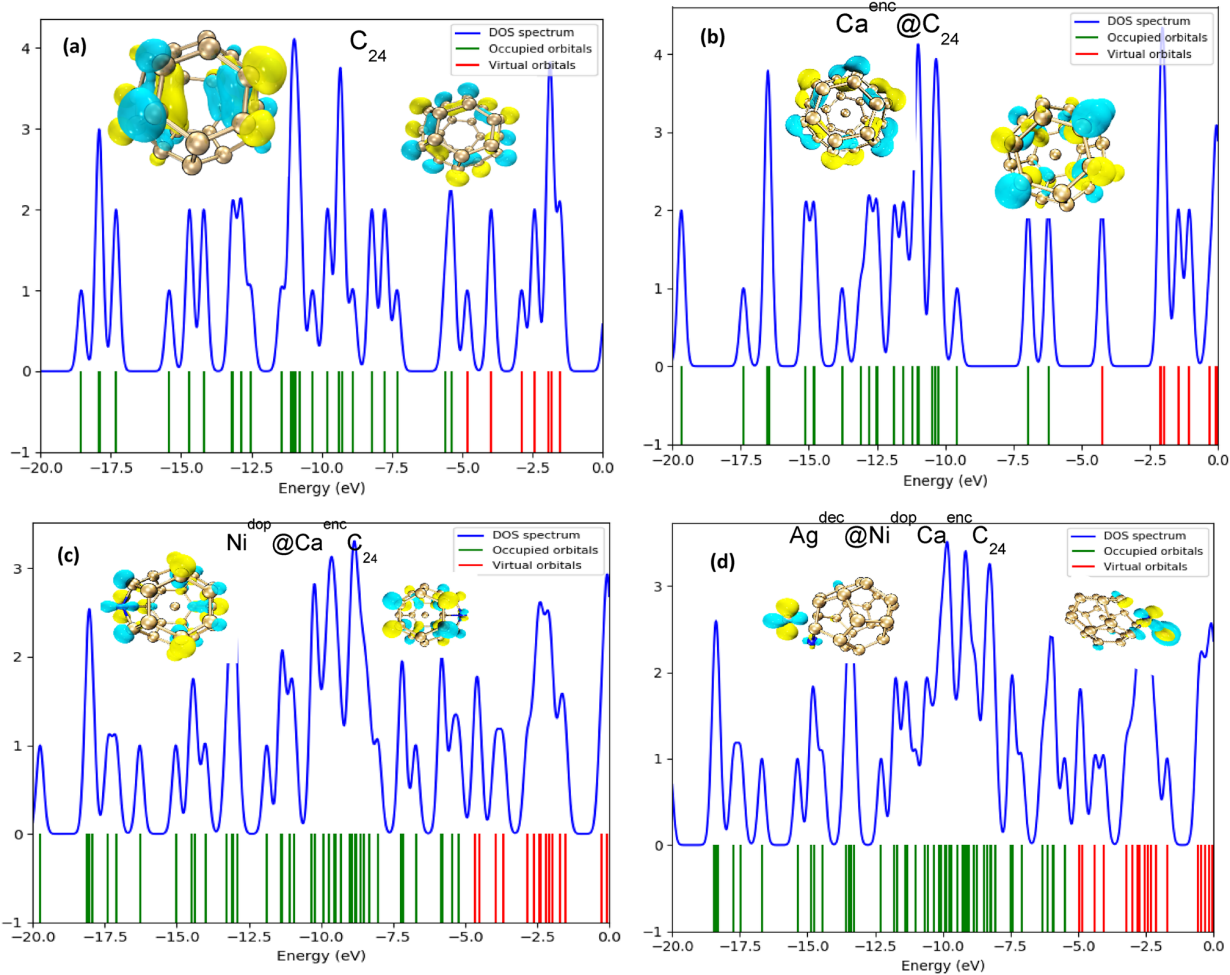
(**a–d**) HOMO and LUMO iso-surface distribution patterns and total density of state (TDOS) mapping of the studied bare systems (S_1_–S_4_).

The density of state plot showing the relevant energy gaps was executed to understand and measure up the changes in electronic properties of the studied engineered systems before and after adsorption of H. The total density of state plot before adsorption of the studied systems were distinctively plotted for better visualization of the difference arising due to the interactions of the surfaces (Fig. 4) with the hydrogen. The changes observed in the total density of state (TDOS) peak intensities, likewise the peak distinguishable shifts reflect the changes in conductivity upon adsorption of atomic hydrogen, over the S_1_-S_4_ surfaces. More observable changes in the TDOS intensity peaks, and energy levels were also observed in the virtual orbitals of the Ag^dec^Ni^do^Ca^enc^C_25_ system; this is in tandem with the reactivity analysis (FMO), where the highest change in energy gap of Ag^dec^Ni^do^Ca^enc^C_24_ was observed due to the significant change in LUMO energy.

Also, the electrophilicity which is related to the chemical reactivity of a compound or system^29^ was also calculated. Thus, the bare studied surfaces (S_1_–S_4_) have electrophilicity index values of 6.0888, 11.9820, 5.9449, 5.2983 eV. More high values are observed as calculated when atomic hydrogen was adsorbed on the titled engineered surfaces, with H@Ni^dop^Ca^en^C_24_ and H@Ag^dec^Ni^dop^Ca^en^C_24_ having the least value of electrophilicity (5.6705, 6.1707 eV), suggesting that our Ni^dop^, Ag-decorated carbon-based structures are best candidate for H-adsorption and electrocatalysts for hydrogen evolution reaction^30^.

#### Electronic structure change (NBO analysis)

Natural bond order (NBO) analysis was employed for this study to gain more insight into concerns of the mechanisms of adsorption. The donor–acceptor interactions in the NBO cadre were extracted and carried out by the second order perturbation theoretical level, which is included in the NBO analysis. With regards to this, energies of delocalization of electrons from occupied NBOs to empty NBOs, e.g., obtaining stabilization energies, *E*(*2*), which was gained by the donation from the donor NBO to the acceptor NBO. Thus, for each respective donor NBO(i) and acceptor NBO(j), the stabilization energy related by *i → j* delocalization is keenly estimated on the basis of the second order perturbation theory as reported elsewhere. The donor–acceptor interactions of the studied systems (with corresponding numbering scheme are given in Fig. 6 between the H-molecule and the fullerene(C_24_) engineered surfaces. In addition, the most important interaction was investigated between the H-atom, and the studied systems, in order to emphasize and know more intrinsic details of their bond nature. Table 4 showed the titled systems stabilization energies for donor, and acceptor orbitals of all systems of interest. In all distinctive cases, the donor orbital emanates from the lone pairs of carbon (C), and Nickel (Ni) from S_1_-S_2_ and H_a_-H_b_ molecules, and acceptor orbitals (j): π*, LP from carbon and nickel atoms obviously from the NBO tabulation (see Table S1-supporting information). The transition observed for the titled molecules majorly showed flow of π → π*, π* → π*, LP(1)* → σ*, whereas for the interactions the transition observed was also of the majority; anti-bonding π orbitals.

Interestingly, as tabulated in Table 4, the order of E2 stabilization energy for the bare surfaces; C_24_, Ca^enc^C_24_, Ni^dop^Ca^enc^C_24_, Ag^dec^Ni^dop^Ca^enc^C_24_, and the various studied hydrogenated systems are relatively high, with the H-adsorption of Ag^dec^ surface exhibiting high stabilization energy, thus agreeing with the observed theoretical computation of adsorption capacity, reactivity of Ag^dec^ to electrocatalytic hydrogen evolution reaction. It can also be concluded from the NBO analysis that the Carbon(C), and Nickel (NI) atom of the studied molecules in S_1_–S_4_, H_a_–H_d_ are stronger donors compared to the other atoms in the molecules. Also, the significance of the chemical interactions which was assessed using the total sum of the delocalization energies (ΣE) could be interpreted according to literature^31^ and explicit studies that larger ΣE connotes those interactions resulting from electron delocalization from donor to acceptor between S_1_–S_4_, H_a_–H_d_ and the adsorbing H-molecule is very essential.

#### Electronic charge transfers analysis (q_ECT_)

Charge transfer interaction can densely occur when we have an electron donor and an electron acceptor site in a molecule that determines donor –acceptor complexes. As such, natural bond order (NBO) instituted an enhanced technique for investigating the bonding interactions within atomic orbitals and between atomic orbitals, providing an insight for studying the intermolecular charge transfer in molecule^31^. Thus, the charge transfers for the studied bare systems and the hydrogenated engineered systems were calculated using the equation^32,33^.

2 $${\text{QECT }} = { }\left( {\frac{{F_{i,j} }}{{E_{i} - E_{j} }}} \right)^{2}$$

where n is the orbital occupancy, ${E}_{i}-{E}_{j}$ are the diagonal elements, and ${F}_{i,j}$ is the off diagonal natural bond orbital Fock matrix element. Considering the above relation, the optimal charge transfers between the adsorbed studied engineered systems (H@C_24_, H@CaC_24_, H@Ni^dec^Ca^en^C_24_, H@Ag^dec^Ni^dop^Ca^en^C_24_) were evaluated and represented in Table S1–S2. The electronic properties which were characterized by NBO and FMO analysis, gave more details to the electronic charge transfer for the studied engineered systems. The highest charge transfer was observed for H@Ag^dec^Ni^dop^Ca^en^C_24_ with charge transfer index of 0.0234é, a d the least charge observed for the H@C_24_. The NBO analysis expressed that highest occupancy was also observing to be exhibited by the Ag-decorated studied system, which is in tandem with the high charge transfer it offers, which is a key responsible factor in its high stabilization energy.

#### Quantum theory of atoms-in-molecule (QTAIM)

**Figure 5:**
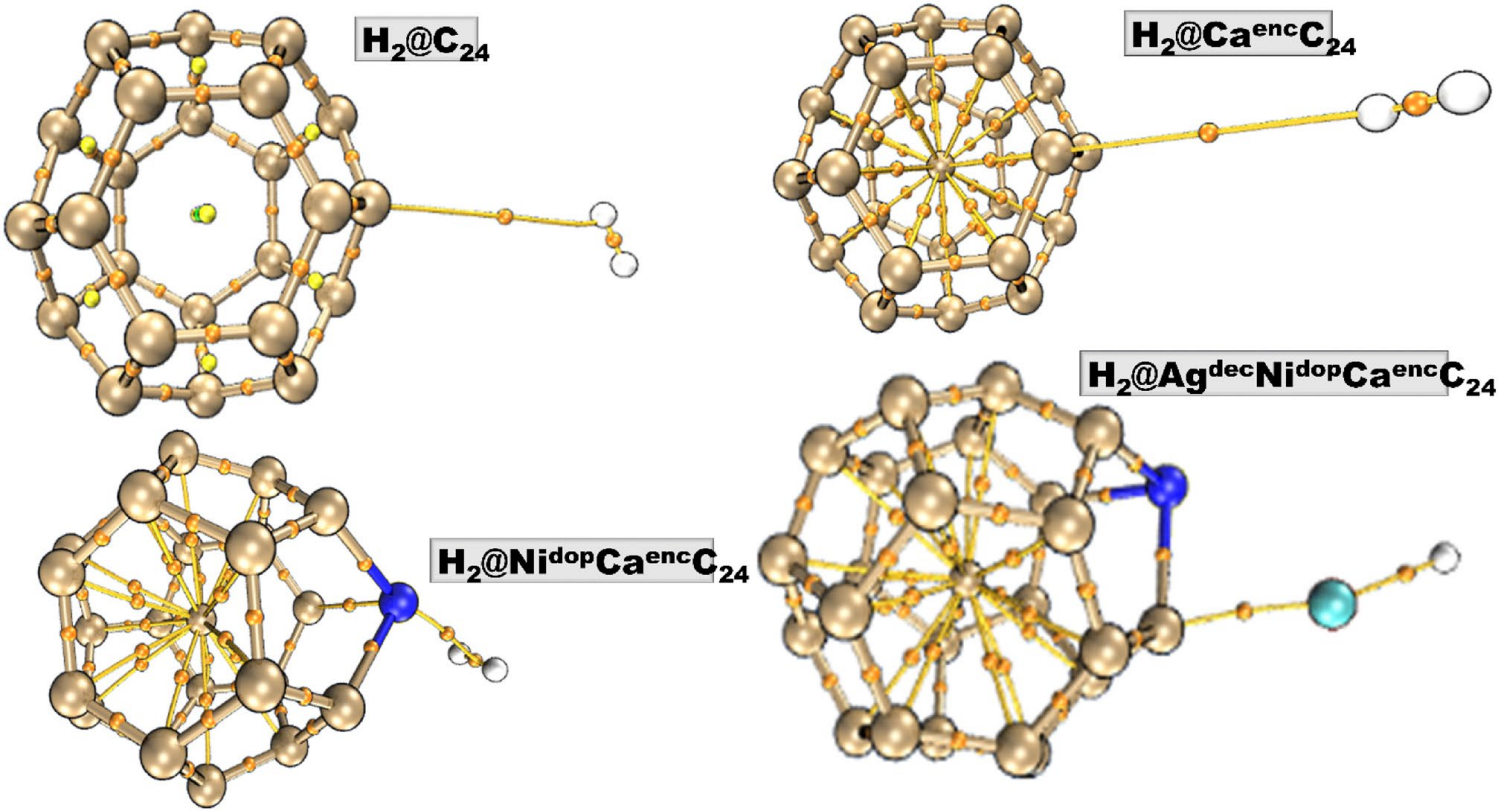
QTAIM molecular graph plot of the studied hydrogen-adsorbed systems.

The Bader’s quantum theory of atoms in molecules (QTAIM) was employed to analyze the non-covalent interactions of the adsorbed hydrogen (H), and the studied systems. The quantum parameters used to expatiate the non-covalent interactions at bond critical points (BCP’s)^34^ in the QTAIM analyses are the Laplacian of electron density $(\nabla$^2^$\rho )$, potential energy density (V(r)), Hamiltonian kinetic energy density (K(r)), Lagragian of electron density (G(r)), density of electrons ($\rho$(r)), and total electron energy density (H(r)). Hence, the values of the extracted bond critical point parameters via QTAIM analyses are given in Table 2, and the iso-surface of the studied systems given in Fig. 5. For enclosed shell interactions (that is the atomic adsorbed hydrogen), the density of electrons ($\rho$), which is the measure of the grand probability of an electron being present at an infinitesimal element of space surrounding any given point^35^.The bond critical point (BCP) ranges from 0.1123 to 0.2664 for the atomic hydrogenated systems (H_a_–H_d_), which implies that some atoms present in the studied systems is apparently shifting negative charges away in the titled structures and thus, expatiating more on the adsorption capacity of the titled systems, as low electron density value from literature study have shown relatively high adsorption capacity and catalytic reactivity^36^. This so implies that for the studied atomic hydrogen adsorbed systems, with H_d_ having the least electron density depicts its good and proficient catalytic ability. Hence, for the enclosed interacted systems of interest, strong electrostatic hydrogen-bond interactions were observed with the contributing atoms majorly dense on the hydrogen and carbon. The molecular adsorbed hydrogen electron density was also observed to be localized on the hydrogen, and the engineered Ni^dop^, Ag^dec^ system, whereas the encapsulated, and the fullerene systems had its electron density distribution on the hydrogen atoms majorly.

**Table 2:** The obtained values of the topological parameters of the BCP’s of the studied hydrogenated systems from QTAIM analyses.

System’s interaction	BOND	$\rho$(r)	$\nabla$^2^(r)	G(r)	K(r)	V(r)	H(r)	G(r)/V(r)	ELF
H@C_24_	H_25_–C_5_	0.2664	− 0.9920	0.3064	0.2786	− 0.3093	− 0.2786	0.9906	0.9907
H@CaC_24_	H_26_–C_20_	0.2685	− 0.1065	0.2635	0.2927	− 0.3190	− 0.2927	0.8260	0.9933
H@NiCaC_24_	H_26_–Ni_25_	0.1363	0.1455	0.9794	0.6158	− 0.1595	− 0.6158	6.1404	0.5284
H@AgNiCaC_24_	H_27_–Ag_27_	0.1123	0.1188	0.7460	0.4489	− 0.1195	− 0.4489	1.6618	0.5027
H_2_@C_24_	H_25_–H_26_	0.2577	− 0.1105	0.5347	0.2762	− 0.2762	− 0.2762	1.9359	1.0000
H_2_@CaC_24_	H_26_–H_27_	0.2577	− 0.1106	0.9668	0.2764	− 0.2764	− 0.2764	3.4978	1.0000
H_2_@NiCaC_24_	H_26_–H_27_–Ni_25_	0.4746	0.2451	0.6323	0.1961	− 0.6519	− 0.1961	0.9699	0.7388
H_2_@AgNiCaC_24_	H_27_–H_28_–Ag_26_	0.4164	0.16122	0.4546	0.5152	− 0.5061	− 0.5152	0.8982	0.9076

More so, $\nabla$^2^$\rho$ BCP values falls within the value range of − 0.1065 to 0.1455 for the adsorbed atomic hydrogen, and between − 0.1105 to 0.2401 for the adsorbed molecular hydrogen systems (H_e_-H_h_). However, $\nabla$^2^$\rho >$ 0 denotes an enclosed shell interaction (ionic, hydrogen bonding), and weak van der waal interactions. Interestingly, the values of G(r), and V(r) always comes out positive and negative, which is in tandem with our studied systems, respectively at all the distinctive BCP’s. it is worth noting that the total electron energy density (TEED), which is the sum of G(r) and V(r) gives corresponding value of H(r), showing the presence of covalency (H < 0)^37^. Hence for the studied systems, both the atomic, and molecular hydrogen adsorbed systems exhibited negative TEED value. Thus, gratifying the covalent nature of the studied systems. Strong hydrogen bond interaction can also be characterized by the values of H(r) and $\nabla$^2^$\rho$^42^, which is exhibited by the titled interacted systems.

In many appreciative cases, $\nabla$^2^$\rho$ >0 < H show the existence of strong hydrogen bonding^37^, and the value of V(r)/G(r) < 1 is explicitly for a close shell interaction while for a shared shell interaction, V(r)/G(r) is denoted to be > 2^38^. This intense fact show that the studied engineered systems exhibited a close shell interaction. Correlational, with regards to the studied systems interaction with hydrogen, three (3) BCP’s were observed each as shown in Table 5 and Fig. 5. Thus, the BCP’s values of G(r), V(r)/G(r) are sure agreement with the electron density, and laplacian of electron density, showing the non-covalent nature of the titled systems. The topological analyses parameters such as electron density, and the laplacian of the electron are in the range of H-bonding. This can be better explained by the long interacting distances (> 1.8) observed from the visualization of the studied systems, which is in tantamount to the H-bonding requirement. These explicit results of the QTAIM analyses are way superior, in that it intensely confirmed some bond interactions that are not possible to be confirmed through structural analysis. Thus, these analyzed results are consistent and in tandem with the theoretical calculated adsorption energies, reactivity studies, and electrochemical calculations of the studied systems, with respect to the engineered encapsulated, doped, and decorated Ca, Ni, and Ag surfaces.

The electron localization function parameters, which is a measure of the likelihood of finding an electron in the neighbourhood space of a reference electron located at a given space and with an individualized spin. When applied to the studied surfaces, an analysis of the ELF shows a clear separation between the participating core and valence electron, and also show an agreement with the studied atom in molecule analysis, analyzing the spin–orbit effects on the electronic structures. The near zero values obtained for the studied interacted systems indicated agreement with the atom in molecules values obtained for the studied modelled systems.

#### Electronic density of state (EDS)

To comprehend the interaction between fullerene(C_24_) engineered carbon-based structure, and hydrogen-adsorbent. It is paramount to study the electronic properties, hence for the purpose, analyses of density of states (DOS) is particularly of keen need. Total density of state (TDOS), partial density of state (PDOS), and overlap partial density of state (OPDOS) (significantly referred to from literatures as crystal orbital overlap population) diagrams were plotted (Figure S1), and analyzed with major interest on PDOS. This was created by entangling the molecular orbital information with Gaussian curves^39^ of heights, and full width of 0.2 eV using multiwave-function software.

The electronic density of state (EDS) is a prerequisite factor in material science that determines properties of metals^40^. It is essentially the number of different states at a distinctive energy level that electrons are allowed to occupy^41^. For a more intense outlook on the effect of the adsorbed hydrogen on the electronic densities of states was analyzed. PDOS was explicitly used for determining the molecular orbital contributions, presenting majorly the composition of the fragment orbitals yielding to the molecular orbitals (Fig. 5) that of all the studied systems, the highest contribution of the frontier molecular orbitals (FMOs) is, for the illustrated concave view, with the adsorption of H-molecule. From the partial density of state (PDOS) of the adsorbed H-plotted in Fig. 4a–d. It can be obviously deduced from the plot that Ag^dec^Ni^dop^Ca^en^C_24_ after adsorbing hydrogen maintains the semiconductivity feature with a narrow energy band gap (0.5279 eV), indicating that the adsorbed hydrogen on the surface of the modelled system contributed majorly to the conductivity of the decorated Ag surface. As such acting as an electron donor, this phenomenon exhibited by the Ag^dec^ system profits from the lower adsorption energy capability comparing with that of the other studied systems. Strong hybridization of orbitals is observed in the PDOS of the Ag^dec^Ni^dop^Ca^en^C_24_ system, hence, the hydrogen atom and nitrogen doped atom contributing minutely to the distribution of electron in the studied referenced system, as depicted in Fig. 4a, which indicated the existence of chemisorption in the adsorption of hydrogen onto the Ag^dec^ surface. In comparison with the adsorption of some intrinsic and modified fullerene(C_24_) nanocage systems^42^ for H-adsorption, the least adsorption energies and a non-significant change in density of state near the activated level implies advantage of C_24_ nanomaterials for electrocatalytic hydrogen evolution reaction. More so, from the graph in Fig. 4a, it can also be wittingly seen that the electron contribution was immensely from the Carbon atoms of the decorated system, followed by the Ag decorated atom. The dotted vertical line demarcating the bonding and anti-bonding orbitals showed the range of HOMO energies, and LUMO energies. Also, it can be stated that the spin-induced magnetic moment resulted in a large decoration effect of Ag, where H performs as an electron acceptor with a large charge transfer (0.365/e/), the bohr magneton parameter (magnetic moment) may also be the reason of maximum adsorption energy and band-gap reduction(0.5279 eV) in the Ag^dec^ system.

In furtherance from the plotted density of state graph for the Ca^enc^ system, partial density of state was also analyzed for the Ca^enc^ surface as shown in Fig. 4, to examine the structural alignment and changes in the electronic properties of Ca^enc^@C_24_ system. In the spatial orientation of H on the Ca@C_24_ system, the first LUMO peak has its density highly sparse on the carbon atom, likewise the second peak as it is followed by calcium atom contributing maximally to the second peak, the HOMO was also observed to have its electron density highly localized on the carbon atom, while the hydrogen atom contributed minutely to the density of the studied system. Also, on adsorption of hydrogen onto the surface of Ni^dop^Ca^enc^C_24_, the contributing atoms which are the carbon, calcium, nickel, and hydrogen. The HOMO, and LUMO is highly dense on carbon, followed by nickel. The band gap observed for the titled system is seen to be narrow which is similar to the other studied systems, considering the electron density and narrow band gap, occurrence of chemisorption between H, and the Ni^dop^Ca^enc^C_24_ can be confirmed. The H adsorption onto this studied system also initiated a strong hybrid orbital in PDOS of the adsorbed hydrogen. In Fig. 4d, it is stated that for the fullerene (C_24_) system, the contributing electron is majorly the carbon atom, with the HOMO, and LUMO site contributing to the energy gap of the titled system, and the partial density of state of hydrogen atom having minute effect on hydrogen invincibly contributing to the electron density of the system. With the observations and the literature assertions on catalytic activities of fullerene engineered nanomaterials, it can be clearly asserted that the iteration of charge density takes place upon the adsorption of hydrogen onto the engineered Ca^enc^, Ni^dop^, Ag^dec^ surfaces. It was also found that, for an ideal metal system the hydrogen atom can stably adsorb at the doping, and decorated-position. On the safer hand, according to density of state (DOS), there is a major contribution of hydrogen molecule to the frontier molecular orbital (FMO), which indicate chemisorption mechanism.

### Electrochemistry studies

#### Hydrogen chemisorption energy

Adsorption energy (E_**a**_) of an adsorbed molecule is the energy, which has to be delivered to the adsorbed atom to be desorbed from the surface. Adsorption is a surface-based exothermic reaction that liberates an expressive amount of energy when gas is adsorbed on a solid surface^43,44^. Hence, declining the residual forces on the surface of the adsorbent. Thus, reducing surface energy. Adsorption energies on average have been calculated for all investigated surfaces for this study in the same manner for hydrogen evolution studies. The various tabulated adsorption energies were calculated using the Gen basis set. The onset of this study is a consistent set of hydrogen chemisorption energies. These were obtained theoretically from density functional theory (DFT) calculations and, all calculations employed the TPSSh meta-generalized- gradient-approximation exchange–correlation functional in calculating transition states and energy barriers^45^. The dipole correction was applied throughout the calculation to take into account the polarization effects of the studied surfaces^46–49^. The adsorption energies of hydrogen atom (${E}_{ads}^{H}$) are defined as the energy difference pre (before) and post (after) the adsorption concerning the gaseous phase H_2_ molecule as shown in the preceding step below:

3 $$E_{ads}^{H} = {\text{ E}}_{{({\text{Total}})}} - {\text{ E}}_{{({\text{Surface}})}} {-}\frac{1}{2}{\text{E}}_{{({\text{H2}})}}$$

where E_(Total)_, E_(Surface)_, and E_(H2)_ are the energies for the interactions, studied surface energies, and energy of the optimized hydrogen molecule in the gas phase and hydrogen atom adsorbed on the studied surfaces, respectively. Hence, the more negative the ${E}_{ads}^{H}$ is, the more rigidly bound and strongly the H atom adheres on the surfaces^50^, corresponding to an exothermic and favorable adsorption process, whereas a positive value indicates an endothermic and unfavorable adsorption process. More so, the larger the E_ads_, the more stable is the surface slab + H_2_.

The adsorption energy results from the various calculated chemisorption energies indicate negative value energies in the order H_2_ > H_3_ > H_1_ > H_4_. This indicates that the H_4_ has the more negative adsorption energy, indicating the interaction that gave the stronger hydrogen adsorption. Thus, having a more decorated, doped, and encapsulated surface for hydrogen adsorption with the adsorption energy of − 0.065 eV. The presence of hydrogen shows a unique and major transference of electron density from the studied surfaces to the adsorbed systems, appreciating the adsorption energies from the clean surface and the adsorbed systems. Thus, contributing maximally to the various adsorption energies of the studied systems as shown in Table 3.

**Table 3:** Referenced adsorption energies (${\Delta E}_{ads}^{H}$) of Fullerene engineered surfaces towards adsorbed hydrogen together with adsorption energies of other carbon-based structures towards studied molecule (H) with corresponding references in square brackets.

Adsorbed molecule	${\Delta E}_{ads}^{H}$(eV)			
Hydrogen (H)	CNT^dop^	Graphene^dec^	C_21_H_12_^dec.^(Sumanene)	C_24_-engineered surfaces
	− 0.182 to − 0.320^46^	− 0.150 to − 0.530^46^	− 0.830 to − 1.44^46^	− 0.137 to − 1.965

Adsorption energies which as obtained in this work together with adsorption energies of other representative carbon-based engineered structures with hydrogen (carbon doped nanotube, graphene-decorated layer, sumanene doped surface) with corresponding references and details are presented in Table 4. The ranges indicated for the aforementioned adsorption energies depends on the probable adsorption sites and calculated level of theory used. For explicit details, one should refer to the cited references. As stated by Peng et al.,^51^, carbon based engineered nanotubes cab neither adsorb nor give a profound catalytic activity with hydrogen molecule. While studies by Gao et al.^52^ and Rikalo et al.^53^ indicated positive adsorption properties of carbon-based engineered magnesium and Sumanene, respectively, towards H-molecule. Gao et al. carried out a density functional theory (DFT) calculation within a plane wave basis set (ABINIT) and invoking generalized gradient approximation (GGA) of PBEO (Perdew-Burke-Ernzerhof) functional, while Rikalo et al.^53^ employed long range exchange Becké, Lyp and Yang Par exchange functional with 6-31G(d) basis set. Our study carried out showed positive results for the chemisorption adsorption properties of fullerene engineered surfaces towards H-molecule, especially when it comes to adsorption of Ag^dec^Ni^dop^Ca^enc^C_24_ surface, of sure the obtained adsorption energy of -1.965 eV, indicating that Ag^dec^ out of the other studied C_24_-engineered surfaces can adsorb H- molecule. The result obtained also show that the studied C_24_-engineered surfaces also show that the C_24_-engineered encapsulated, doped, and decorated surfaces has much better adsorption energies than graphene-engineered surfaces, which are in most cases comparable with adsorption energies of fullerene (C_24_) surfaces towards H-molecule. Results obtained from Gao et al.^54^, using Moller–Plesset second order perturbation theory (MP2) level of theory with basis sets ranging from 6-31G* to augmented-correlation consistent polarized valence triple zeta (aug-cc-pVTZ) basis set. Without counterpoise correction the results obtained for the studied engineered carbon-based structures (− 0.137 to − 1.965 eV), respectively indicate adsorption properties comparable with carbon-nanotubes, and exhibited better adsorption properties than the graphene and sumanene engineered surfaces.

**Table 4:** Calculated atomic, molecular adsorption energies, co-adsorption energies (${\Delta E}_{ads}^{H}$,${\Delta E}_{ads}^{H2}$ (eV)$,{\Delta E}_{co-ads}^{H-H}$), free adsorption energies, and Energy barrier for the formation of H_2_ by photocatalytic reaction mechanism on the different studied surfaces.

Models/surfaces	${\Delta E}_{ads}^{H}$ (eV)	${\Delta E}_{co-ads}^{H-H}$ (eV)	${\Delta E}_{ads}^{H2}$ (eV)	${\Delta G}_{H}^{*}$ (eV)	Energy barrier (eV)
H_2_@C_24_ (H_a_)	− 1.809	15.00	− 16.314	− 1.069	4.57
H_2_@Ni^dop^*Ca^enc.^C_24_ (H_b_)	− 0.137	− 14.18	− 16.587	− 0.103	1.93
H_2_@Ca^enc.^C_24_ (H_c_)	− 1.463	14.55	− 28.255	− 1.223	2.42
H_2_@Ag^dec^*Ni^dop.^Ca^enc.^C_24_(H_d_)	− 1.965	− 15.33	− 16.470	− 1.575	0.82

Unit of conversion (1 Hartree = 27.211 eV); dop = doped, enc. = encapsulated, dec. = decorated.

For the study of graphene-engineered systems towards hydrogen (H), Lee and Kim used density functional theory (DFT), long range corrected (LRC); (CAMB3LYP), and MP2 approach with 6-31G** basis set. On the other hand, for the crystal investigation of Sumanene adsorption properties towards H-molecule. Rikalo et al.^53,55^ used the split DFT theory with both GGA and LDA approximations using PBEO functional, respectively, together with polarization atomic orbitals as basis set. For the Sumanene surfaces, adsorption energies ranging from − 0.830 to − 1.440 eV was obtained respectively, which is comparably better than other referenced engineered carbon-based illustrated nanostructures. Hence, the engineered fullerene structures still remaining the adsorber of H-molecule. Hence, the studied fullerene (C_24_) engineered surfaces (ranging from C_24_ → Ag^dec^Ni^dop^Ca^enc^C_24_ exhibited the best adsorption properties for hydrogen molecule as in the case of adsorption energies obtained tends to be much higher than the adsorption energies of other referenced carbon-based engineered systems towards hydrogen-molecule.

### Adsorption phenomenon

#### Atomic hydrogen adsorption

This section presents results concerning the structure of fullerene carbon-based engineered structures (CBES) after the adsorption of hydrogen. Figure 2 illustrates the distances between the various studied CBES and the adsorbed hydrogen after geometrical optimization together with intramolecular distances on the adsorbed atomic hydrogen. Single hydrogen atom can be initiated universally with respect to microstructures and nanocages, by manufacturing operations which environmental cleaning is not left out^52^, as well as environmental exposure at low temperatures and gaseous hydrogens at an elated temperature. Thus, when a single hydrogen atom is adsorbed on a metal system, it can easily penetrate into the lattice, depending on the conditions the adsorption process is exposed. Thus, owing to the small size of hydrogen atom, hydrogen atom can disperse interstitially and do not require a place exchange phenomenon as the case of oxygen penetration on metal-systems. Hence, this penetration of hydrogen atom on metal- clean surfaces can undermine the stability of the metal lattices. The outcome of this phenomenon can as much leas to embrittlement of hydrogen, that can lead to the degradation of the metal-structures and adsorbed hydrogen systems.

**Figure 6:**
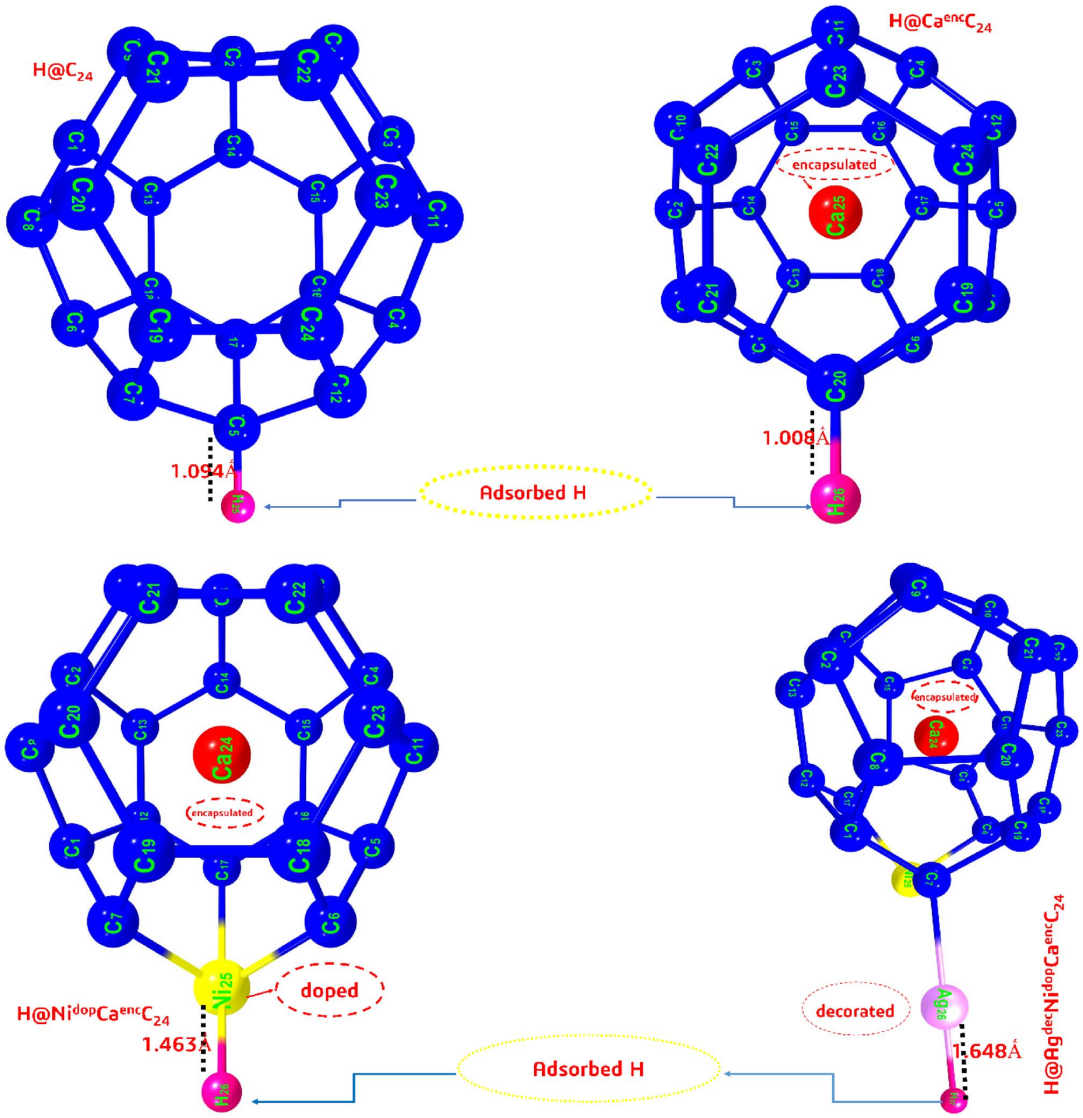
Structural illustration of the titled systems on adsorption of atomic hydrogen, with their respective bond lengths.

More so, hydrogen adsorption with keen interest on atomic hydrogen adsorption also affects the adsorption characteristics of metal-surfaces and adsorbed systems of contact, as much decreasing the bond-strength interaction between the clean surfaces and post adsorbed systems comparatively. This is in relation with the studied metal-surfaces and the fullerene structure, whose adsorption capacity decreases after they have been adsorbed by the single hydrogen atom, hence initiating an increase in bond-lengths of the studied systems (Table 5). The H-Ni bond length increases with increase in Ni doping in the range of H@NI^**dop**^Ca^en^C_**24**_ < H@Ag^dec^Ni^**dop**^Ca^en^C_**24**_, having the bond length of 3.695 Ấ, and 1.648 Ấ respectively. Also, an increase in bond length for the encapsulated systems was observed to have a H-Ca increment in angstrom bond, in the order; H_**d**_ > H_**b**_ > H_**c**_ with bond lengths of 6.264 Ấ, 3.920 Ấ, 3.490 Ấ respectively, and the decorated systems forming moderate chemisorption H–Ag bond length of 1.648 Ấ. Hence, it was critically observed that an increase in Ni-doping, Ca-encapsulation, and Ag-decoration gradually increases the adsorption energy, bringing about more negative value of the adsorption energy. More so, literature and scientific assertions has showed that the greater/high negative value the adsorption energy, the stronger the bond between H and the catalyst systems, and at such more stable the system^55^. More so, the most craving site for an atomic hydrogen on the studied systems were also analyzed. The adsorption sites for hydrogen atom on the C_**24**_(fullerene) surface was on carbon 5(C_**5**_) of the fullerene hollow sphere, at the side of the C_**5**_ (SC_**5**_), as each of the carbon on the fullerene sphere have equal probability of adsorbing atomic hydrogen on any of the carbon surfaces. However, the adsorption site of hydrogen on the doped Nickel (Ni) atom on H_b_ and with a side-view of configuration. Hence, the stable configuration of the titled hydrogen adsorbed systems, with Calcium (Ca) encapsulation, and Ag decoration, with adsorption site taking side- view configurations. Thus, when a hydrogen atom is being adsorbed on the studied systems, the structural optimization projects that the atomic hydrogen moved closer to the doped and decorated Ni and Ag atoms, as such resulting to a shorter bond distance of 1.464, and 1.648 Ấ, respectively for the titled H_b_ and H_d_ systems. It is paramount to take note that, while atomic hydrogen was adsorbed on the studied hollow systems as shown in Fig. 6, with their respective bond distances, no obvious distortion was observed from the projected systems.

**Table 5:** Hydrogenated studied systems, and the electronic population of atomic hydrogen at different adsorption sites.

Atomic hydrogenated system	Bond interaction type [d_H-Atom_]	Adsorption site
H@C_24_ (H_a_)	H → Ca	C_5_.F (side)
H@Ni^dop^*CaC_24_ (H_b_)	H → Ni	Ni_25_.F(side)
H@CaC_24_ (H_c_)	H → Ca	C_20_.F (side)
H@Ag^dec^*NiCaC24 (H_d_)	H → Ag	Ag_26_∙C_7_.F (side)

#### Molecular adsorption

**Figure 7:**
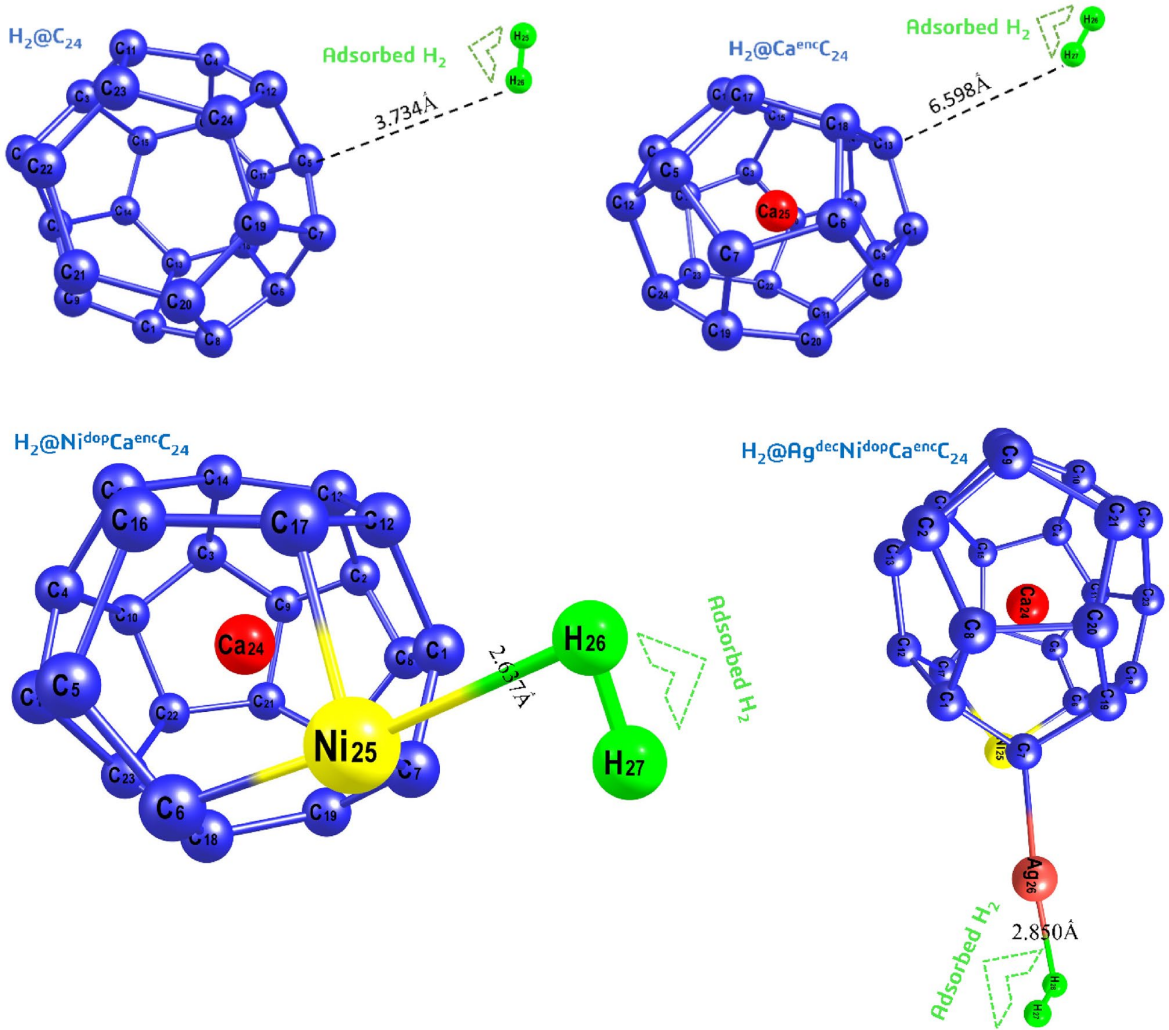
Structurally optimized structures of molecular hydrogen adsorption on the considered nanoclusters exhibiting the bond length of interest.

Aftermath, the adsorption of two hydrogen atoms per surface of the systems studied was considered, it was profoundly observed that the hydrogen molecule was adsorbed with energies of − 16.314, − 16.587, − 28.255, and − 16.470Ấ for H_a_, H_b_, H_c_, and H_d_, respectively, with no distortion in the various structures as shown in Table 4. As shown in Fig. 7, the bond distance H_2_–Ni, H_2_–Ca, H_2_–Ag; d_H2–Ni_, d_H2–Ca_, d_H2–Ag_ summarizing to 2.637, 6.598, 2.850 Ấ for the systems of interest. The adsorption of H_2_ in the studied systems were obviously observed to have stronger adsorption energies^56–58^ with extensive negative values, indicating more intense adsorption capacity. The adsorption capacity of molecular hydrogen in the studied systems is in the order; H_c_ > H_d_ > H_a_ > H_b_. It was observed that the hydrogen molecule approaches the side view location of the titled doped, and decorated systems. However, for the encapsulated systems, the hydrogen molecules can take an ectopic adsorption conformation, this simply explain that the fullerene surface engrossed with carbon atom exhibit stable conformation where the molecular hydrogen can be adsorbed on different sites. More so, here in molecular adsorption energy have a significant impact on the hollow structure stability, that is to say that the lower the adsorption energy, the better of the systems’ stabilization. The molecular adsorption energy is defined as^59^;

4 $${E}_{ads={E}_{H2/surface}}-\frac{1}{2}{E}_{H2}-{E}_{surface}$$

where E_H2_ is the theoretical is the output energy of H_2_, E_ads_ is the binding energy of the adsorbed atoms of the titled systems, E_H2/surface_, is the total energy of the system with adsorbed hydrogen molecule, and E_surface_ is the energy of the adsorbed surface, respectively. Where the negative values of the adsorption energies calculated indicated an exothermic adsorption. Thus, molecular adsorption leads to accumulation of negative charge at the corresponding adsorption sites, depending on the interaction between the adsorbed H_2_ and the systems of interest^60^.

#### Chemisorption dissociative adsorption

Dissociative adsorption is one of the most prominent chemical reactions at surfaces. Since, it involves the cleavage of a strong covalent molecular bond, and at the same time, the formation of new chemical bonds of atomic nature. Foremost, the adsorption of H_2_ molecule on the studied C_24_, Ni^dop^Ca^en^C_24_, Ag^dec^Ni^dop^Ca^en^C_24_ was analyzed, and oriented to their surfaces and being adsorbed. The split dissociated hydrogen adsorbed on the hollow surfaces of the titled systems were also investigated as shown in Fig. 4. These theoretically calculations have been computed as follows; a molecule of H_2_ was fixed adjacently on z-axis, to the studied surfaces at 2.962, 2.524, 1.813, and 3.864 respectively for the titled Ag^dec^Ni^dop^Ca^en^C_24_, C_24_, Ca^enc.^C_24_, and Ni^dop^Ca^en^C_24_ surfaces. It was obviously observed from Fig. 4 that the molecular dissociation of hydrogen was close to the surfaces. Hence, the adsorption sites that interacts with the atoms of the studied molecules. This considered adsorption sites makes H_2_ dissociate readily, promoting each hydrogen atom without any structural distortion. However, no distinctive difference among adsorption energies in the final stable configuration of dissociative adsorption on the hollow sites was observed.

More so, it is of paramount to emphasize that, the bounded molecular state described, ought to be considered judiciously. Hence, the exchange correlational basic functional used does not in any way contribute to the right modelling accounting for the van der waal interactions, and as such might not provide an accurate description of such weakly bounded state of the dissociation^29^. The hollow adsorption site therefore was used for the calculation of the surface reactions and energy barriers of the studied H_2_ adsorbed systems.

#### Gibb’s free energy

Photocatalytic H_2_O splitting has turned to be one of the crucial reactions that is divided into two divisions; oxygen evolution reaction (OER) and hydrogen evolution reaction (HER). Hence, the standardized HER taking place at the electrode in contact with the electrolyte, consisting of two reaction mechanics; Volmer Hyrovsky, and Volmer Tafel in protonated media. Regardless, of the pathway followed by HER, both mechanics revealed that the adsorption energy of hydrogen atom plays a distinctive role in determining the catalytic activity on the referenced catalyst surfaces. The energy of adsorption of H_2_ concurrently is related to the adsorption Gibb’s free energy (ΔG_H_) of H_2_ on the catalyst surfaces, and is widely accepted to be a pin interest and mapped description for a catalyst towards HER activity. Hence, more concise explanation on the implications of the adsorption free energy as an energy descriptor for HER is provided in ref.^61^.

To lucidly evaluate the catalytic activity at different hydrogen adsorption, differential Gibb’s free energy for hydrogen adsorption is computed as;

5 $$\Delta {G}_{H}= {E}_{ads}-\Delta {E}_{ZPE}-T\Delta S$$

where ${E}_{ads}$ is the hydrogen chemisorption energy of the titled systems, and $\Delta {E}_{ZPE}$= the zero-point energy difference between the adsorbed and gas phase of H_2_, with its value ranged at 0.04 eV at room temperature condition. Hence, taking the value as a representative value for the studied systems, and $\Delta S$ is the entropy change of the system, with the corresponding standard entropy correction value of − 0.20 eV. According to literature; Norskov^62^ simplified Gibb’s free energy equation, which is widely used in theoretical research fields of HER in calculating free energy. Hence, is stated as;

6 $$\Delta {G}_{H}= {E}_{ads}+0.24 \mathrm{eV}$$

**Figure 8:**
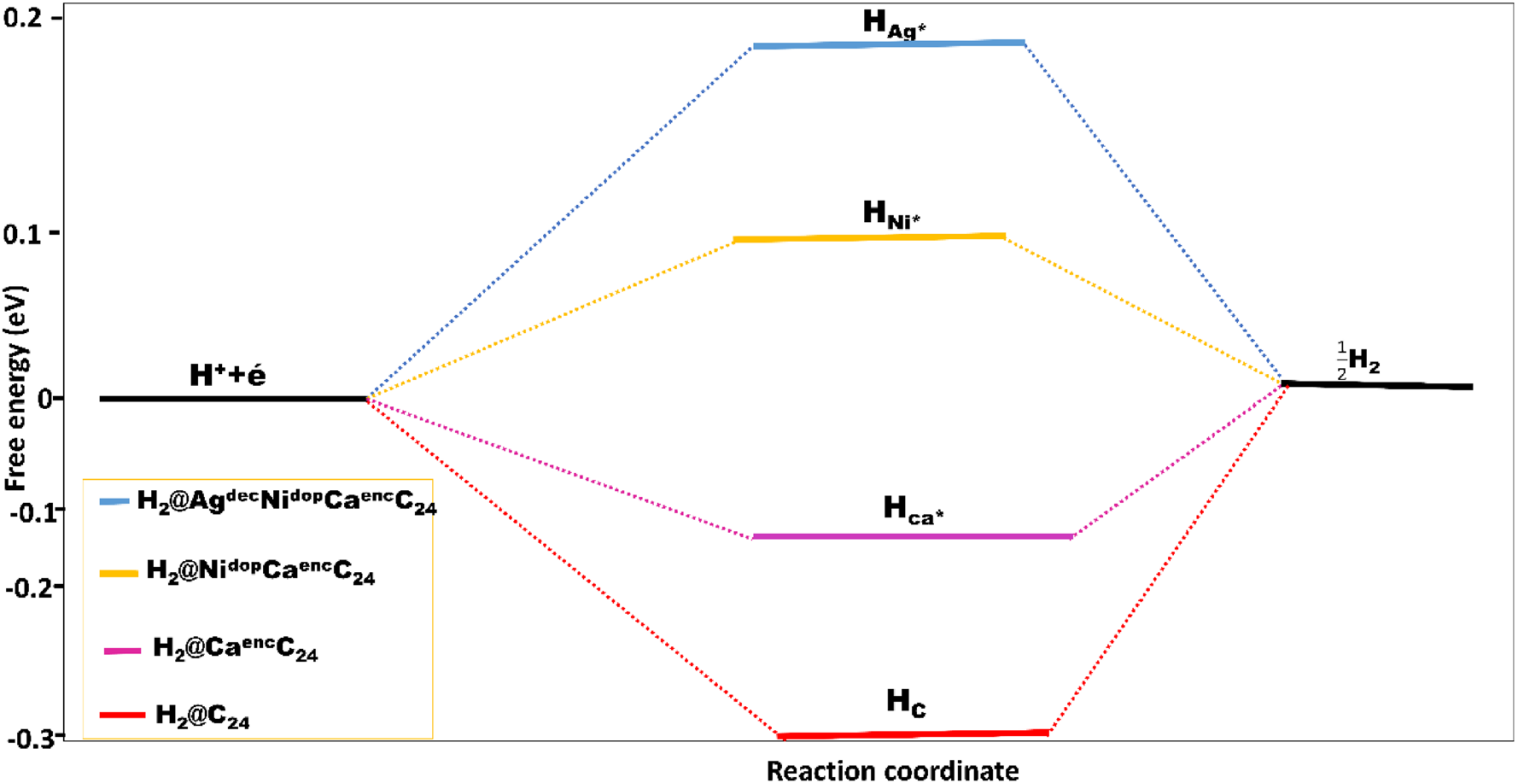
The Gibb’s free energy ($\Delta {G}_{H}$) volcano-curve of the least to highest of the best adsorbed modeled systems (H_c_ < H_Ca*_ < H_Ni*_ < H_Ag*_).

For this research, the studied system’s free energy was calculated optimally using Eq. (3), of which the $\Delta {G}_{H}$ values were computed and tabulated in Table 1, the studied hydrogenated molecules exhibited an intrinsic free energy value of negative, indicating an optimal HER potential catalytic surfaces, and that Hydrogen (H) is softly adsorbed on the surfaces, but difficult to desorb. As the computed Gibb’s free energy were in ascending negative or near zero value of H_b_ > H_c_ > H_a_ > H_d_, with the decorated hydrogen system; H_d_ having the more least near zero value, with $\Delta {G}_{H}$ value of -0.075 eV, and as such possesses a distinctive catalytic surface for HER. Thus, for an optimal HER catalytic systems, $\Delta {G}_{H}$ should be probably be zero or of more negative value^8^. It is also worthy to note that a very strong negative value of $\Delta {G}_{H}$ indicates a very strong hydrogen binding with the studied potential electro-catalytic surface, and with close to zero maximally, the hydrogen adsorption reaches an optimal energy level, hence maximizing HER activity^63^ (Fig. 8).

#### Surface model

The electron transfers to the studied systems, and the formation of surface H atom (H^+^  + é → H_ads_), following the Tafel mechanism, HER can occur. Hence, H_ads_ + H_ads_ → H_2_ (this is priory a surface reaction with zero net charge^64^. Thus, for hydrogen evolution on the titled system surfaces, the Gibb’s free energy (ΔG) is universally taken as the referenced descriptor; wherewithal catalytic sites with too strong endothermic ΔG_H_ is bound to produce low HER activity^54^. More so, for the studied encapsulated, doped, and decorated H_a_–H_d_ surfaces, in relation to the extended surfaces, and stable conformations, there are many possible H adsorption sites on the titled surfaces (Fig. 1). More so, considering the volcano curve^65^ of ΔG_H_ activity relation, the ΔG_H_ on each of the studied systems have been determined on each adsorption site. The Co-adsorption energy of molecular hydrogen (${E}_{ads}^{H-H}$) atoms, was also determined.

In furtherance, the four hollow structures of the studied systems showed average ΔG_H_ approaching the volcano peak (ΔG_H_ = 0 eV) or exhibiting more negative entity, manifesting themselves as a very good catalyst for catalyzing HER. Hence, H_d_ expected to be much lesser than the other doped and encapsulated systems^66^, as its ΔG_H_ value is far from the peak position. And, at the activated complex state, as shown in Fig. 4a–d, the two H-atoms were adsorbed at a stable conformation off-top the adsorbed surfaces with their bond distances stretched from 1.464 to 1.502Ấ in Ni–H bonds, 1.648 to 1.671Ấ, for the doped and encapsulated systems.

Hence, the computed energy barrier E_b_ of 4.57, 1.93, 2.42, and 0.82 eV on the surfaces of H_a_-H_d_, respectively^67,68^. The hydrogen evolution reaction activity on the Ni^doped^, Ca^enc^, and Ag^dec^. Surfaces is efficiently enhanced. More profoundly, from the calculated energetics (ΔG_H_, and E_b_), it could be concluded that the H–H co-adsorption activity on the studied hollow studied structures of C_24_, Ni^dop*^Ca^en^C_24_, Ca^enc.^C_24_, Ag^dec*^NiCa^en^C_24_, following the adsorption order of H_d_ > H_b_ > H_c_ > H_a_. The computed energy barrier in Table 1 is calculated by employing the preceding equation;

7 $${\text{Energy}}\,{\text{barrier}}\,\left( {{\text{E}}_{{\text{b}}} } \right) = {\text{E}}_{{{\text{TS}}}} - {\text{E}}_{{{\text{IS}}}}$$

where ETS is the energy of the various studied systems’ transition state, and EIS attributed to the initial /ground state before transition state optimization.

#### Activated complex of adsorbed hydrogen on C_24_, Ni^dop*^Ca^en^C_24_, Ca^en^C_24_, and Ag^dec*^Ni^dop^Ca^en^C_24_

**Figure 9:**
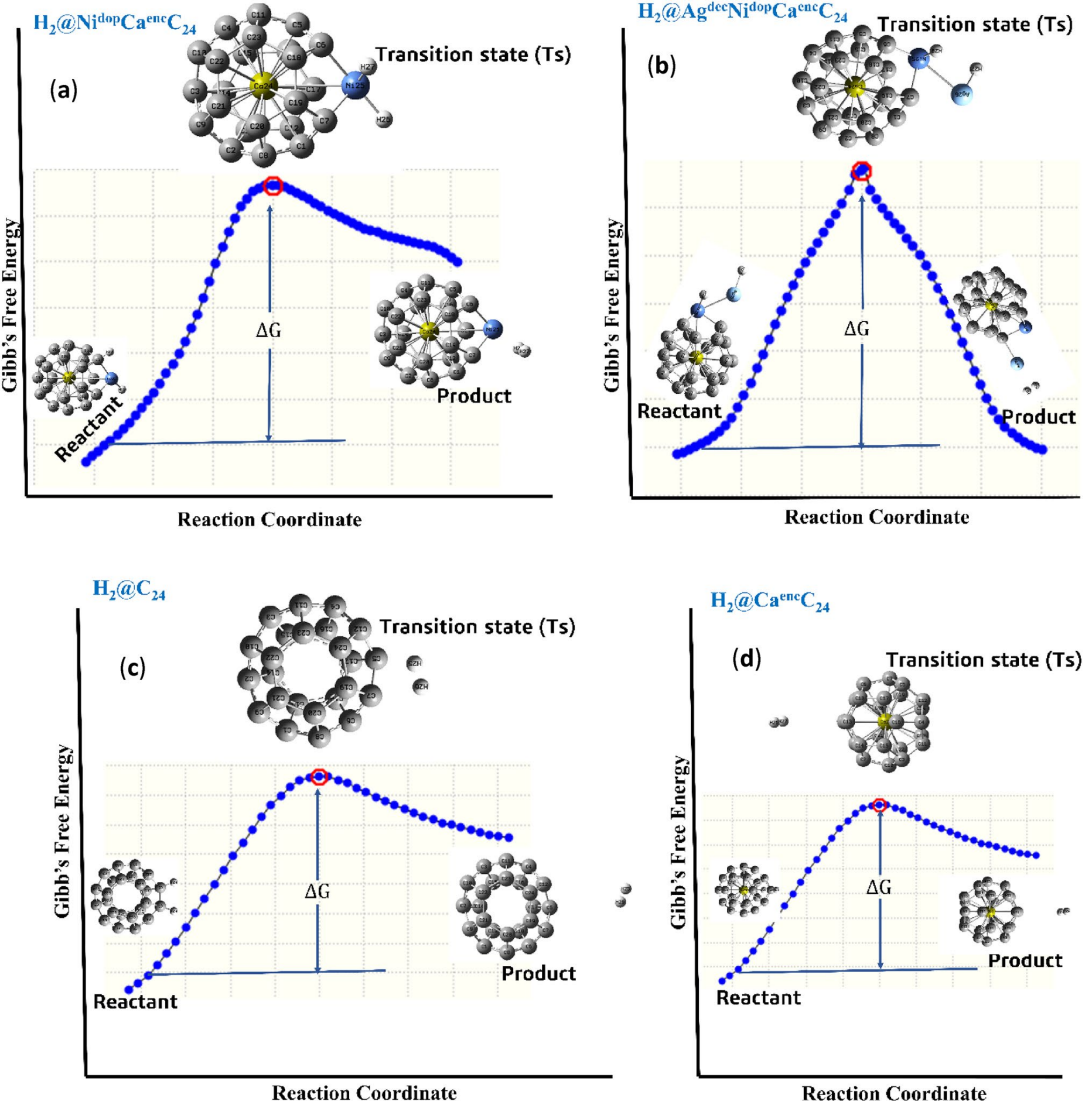
(**a–d**) Absolute energy profile diagram of the titled systems, showing the various transition states on H_2_ adsorption.

An energy barrier is regarded as a potential field that can act as a criterion to either localize or regulate the transfer of charged particles (for example: electrons)^69^. Hence, an energy barrier must be overcome to initiate the transportation of charges released through electron donor (oxidation) and the electron acceptor (reduction) reaction on the anode, thus known as activation energy^70^. As illustrated in Table 6, transferring adsorbed structures into physiosorption state is a prerequisite to reducing energy consumption in the H–H desorption processes. For the purpose of this study, investigating transition processes is essential to regulating surface properties of the engineered encapsulated, doped, and decorated Ca, Ni, and Ag surfaces, because different numbers of the studied tabulated systems can lead to different transition structures with variables transition states energy barriers. In this study, all transition states calculations were conducted for the studied engineered systems, this instills the fact that chemisorption structures only exist in the engineered situations. Thus, the relevant adsorption structures, and energies are shown in Table 6, respectively. More so, with respect to Fig. 9 and Table 4, there are major interesting points worth giving an intrinsic attention. Which include, the transition state barrier of the engineered atoms of interest. In light of structural symmetry, the various engineered surfaces are in fact identical (if the number of encapsulated, doped, and decorated) atoms are overlooked. This clearly states that, effects of the number of encapsulated, doped, and decorated atoms on transition processes are minimal while the most essential factor determining transition processes and the H–H transfer path of transition process is categorically a weak factor.

**Table 6:** Activated complex barrier energy in adsorption processes of the studied systems.

Systems	Transition	Energy barrier (eV)
H@C_24_ (H_a_)	(on C7, andC5)—top view	4.57
H@Ni^dop^*CaC_24_ (H_b_)	(on Ni25)—side view	1.93
H@CaC_24_ (H_c_)	(on C1, and C13)—side view	2.42
H@Ag^dec^*NiCaC24 (H_d_)	(on Ag, and Ni)—side view	0.82

More so, in these transition state processes, the engineered adsorption sites and the carbon (C) sites is the destination of transition process, because of suitability of H–H adsorption on the various adsorption sites of the studied systems. However, it can be concluded that with the encapsulation, doping, and decoration of Ca/Na/Ag atoms, H–H adsorption energies on the various stable adsorption sites changes in maximal range. This feature therefore, elucidates that the adsorption sites (C, Ni, Ag) can be as much regulated to have a desirable H–H adsorption energy by adjusting the engineered elements, which could guarantee efficient electro-catalytic HER as well as low energy consumption during dissociation.

#### HER mechanism

**Figure 10:**
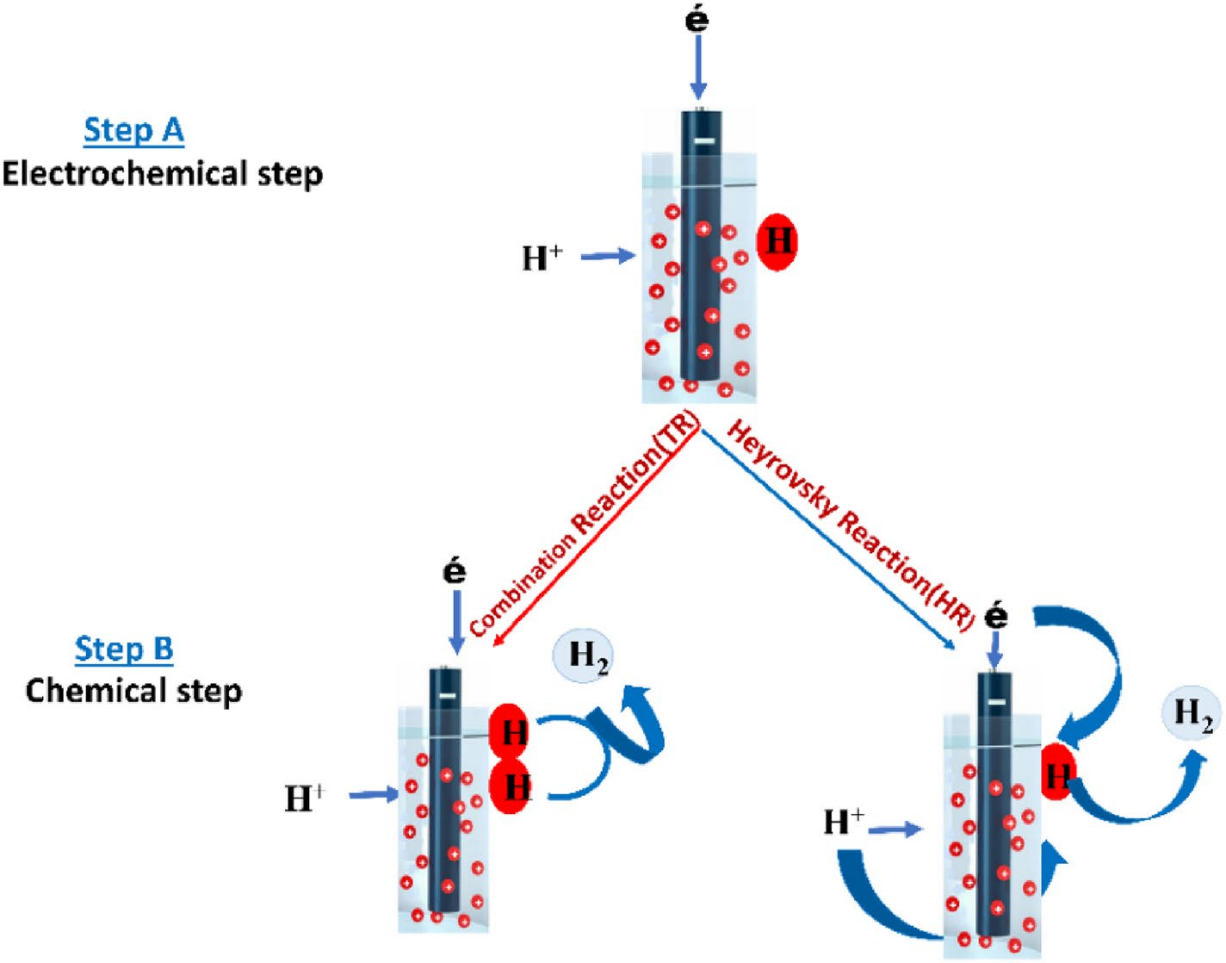
Hydrogen evolution reaction mechanism of reaction on the surface of a conducting pole (electrode).

The evolution of hydrogen is magnificently based on the desorption of molecules, which comes from the positive electrode surface (cathode). Thus, hydrogen evolution apparently occurs on metals, which is a multi-step process taking place on the surface of an electron conducting pole (electrode)^62^. In this sequence, when an electron is being transferred to the electron conducting pole, it is coupled to a positively charged atom (proton) adsorbing on an unoccupied active site of the conducting pole (electrode) to give rise to an absorbed hydrogen atom. Hence, in an alkaline medium, the electrolyte of interest is the water (H_2_0) molecule; whereas the proton source is the hydroxonium cation (H_3_0+) in an acidic electrolyte. As such, in an acidic medium, the discharge of water is implausible^70^. Consequently, molecular hydrogen formation may likely occur through two dissimilar reaction pathways. In the other hand, the transfer of a subsidiary electron to the absorbed hydrogen atom (H) is matched up to the transfer of another proton of interest from the solution of interest, to give off (evolve) H_2_. This process is illustrated with (blue) arrow in Fig. 10 and at such called ion + (Heyrovsky) reaction. In another likelihood, which was attested to for the studied engineered encapsulated, doped, and decorated fullerene-metal systems. On combination, two absorbed hydrogen atoms interacted on the surface of the conducting pole (electrode) to evolve H_2_, hence, this chemical phenomenon is attributed to the “combination or Tafel reaction” (this is shown by the red arrow in Fig. 10). The Tafel slope is predominantly used to show the efficiency of an electrode on its production of current (I) in response to change in applied potential, with regards to HER mechanism^71^. Tafel slope also, clearly indicate the energy potential difference necessary to enhance or decrease the current density by 10 times its initial density. Using the Erdey-Gruz-Butler-Volmer equation, the Tafel slope have been derived theoretically for two distinct limiting cases in describing the electrode kinetics^64^; the low overpotential region (polarization resistance), the high- overpotential region; where the Butler-Volmers equation simplifies the Tafel equation. Thus, Fig. 10, shows the extent of hydrogen evolution taking place through an absorbed hydrogen intermediate. Hence, the activity of the proposed studied potential electrocatalyst in HER is deemed on lowering the energy barrier of any electrochemical reaction. In turn, lowering the electronic potential at which the reaction involved will occur.

## Conclusions

This study employed the meta-GGA functional (TPSSh) with the Gen basis set to study the electronic and electrochemical properties of engineered fullerene (C_24_) nanostructured material. The surface has been engineered by encapsulation with Ca atom, doped with Ni, and decorated with Ag-metal for the enhancement of electro-catalytic hydrogen evolution activity. In order to understand in detailed the electro-catalytic activities of the studied engineered systems, and their effects on atomic and molecular hydrogen adsorption, several electronic and surface properties have been considered to effectively arrive at the various conclusion. The overall results indicates that engineering via a combined approach of encapsulation, doping and decoration enhanced considerably the catalytic activity of the surface towards HER. The computed Gibbs free energy of the engineered systems were found to be closer to zero (− 0.075) thus, affirming the ideal behaviour for enhanced HER activity. More so, the electronic properties based on the NBO and FMO analysis were the kingpins for characterization. For the engineered C_24_ systems and hydrogen adsorbed systems, the HOMO–LUMO orbital electron densities were intensely shifted to the doped and decorated atoms which in turn launched an effect on the E_gap_ of the systems, causing a meagre decrease in the energy gap after atomic and molecular hydrogen adsorption. The Gibbs free energy descriptor and other electrochemical properties all points the Ag^dec^Ni^dop^Ca^en^C_24_ system and its hydrogenated counterparts as the preferred surface model for better electro-catalytic property. Thus, these findings can be potentially useful in predicting C_24_ with Ca, Ni, and Ag (encapsulated, doped, and decorated) systems as electro-catalyst for hydrogen evolution reaction (HER) (Table 7).

**Table 7:** Summary of the tabulated calculated key factor results for electrocatalytic hydrogen evolution reaction modeling.

Systems	Adsorption energy (${\Delta E}_{ads}^{H}$ (eV))	${\Delta G}_{H}^{*}$ (eV)	E_H–L_ gap (eV)
C_24_	–	–	0.5549
Ca^enc^C_24_	–	–	1.9650
Ni^do^Ca^enc^C_24_	–	–	0.5628
Ag^dec^Ni^do^Ca^enc^C_24_	–	–	0.4882
H@C_24_	− 1.809	− 1.569	0.9878
H@Ca^en^C_24_	− 0.137	− 0.103	2.0980
H@Ni^dop^Ca^en^C_24_	− 1.463	− 1.223	0.4661
H@ Ag^dec^Ni^dop^Ca^en^C_24_	− 1.965	− 0.075	0.5279

## Supplementary Information

Supplementary Information.

## Data Availability

All data generated or analysed during this study are included in this article and its supplementary information file.
