# Supplementary material for: Electrocatalytic activity of metal encapsulated, doped, and engineered fullerene-based nanostructured materials towards hydrogen evolution reaction
Source: Sci Rep. 2022 Sep 16;12:15608. doi: 10.1038/s41598-022-20048-3 (PMC9481569; doi:10.1038/s41598-022-20048-3)
Supplement: Supplementary file 1 — Supplementary Information. [file 41598_2022_20048_MOESM1_ESM.docx]

**Supplementary Information**

**Electrocatalytic activity of metal encapsulated, doped, and engineered fullerene-based nanostructured materials towards hydrogen evolution reaction**

**Hitler Louis ^a,b,^ Onyinye J. Ikenyirimba ^a,b^, Tomsmith O. Unimuke ^a,b*^, Gideon E. Mathias ^a,b^, Terkumbur E. Gber^a,b^ and Adedapo S. Adeyinka^c*^**

^a^ Computational and Bio-Simulation Research Group, University of Calabar, Calabar, Nigeria

^b^Department of Pure and Applied Chemistry, University of Calabar, Calabar, Nigeria

^c^Research Centre for Synthesis and Catalysis, Department of Chemical sciences, University of Johannesburg, South Africa

**^*^Corresponding author’s email**: [ojtomtsm@gmail.com](mailto:ojtomtsm@gmail.com) and [aadeyinka@uj.ac.za](mailto:aadeyinka@uj.ac.za)

**Table1S:** Natural bond order analysis table

| MOLECULES | TRANSITIONS | DONOR NBO (i) | ACCEPTOR NBO (j) | E(2) kcal/mol | E(j)-E(i) a.u | f(i,j) a.u |
| --- | --- | --- | --- | --- | --- | --- |
| C | π $\to$ π^*^ | πC_13_-C_14_ | π^*^C_17_-C_18_ | 14.55 | 0.27 | 0.057 |
|  |  | πC_22_-C_23_ | π^*^C_20_-C_21_ | 14.45 | 0.27 | 0.056 |
|  |  | πC_15_-C_16_ | π^*^C_17_-C_18_ | 14.46 | 0.27 | 0.056 |
|  |  |  |  | **𝝨E(2)=43.46** |  |  |
|  |  |  |  |  |  |  |
| Ca^enc^C | π^*^ $\to$ π^*^ | π^*^C_20_-C_21_ | π^*^C_1_-C_8_ | 58.86 | 0.03 | 0.057 |
|  |  | π^*^C_22_-C_23_ | π^*^C_3_-C_10_ | 56.92 | 0.03 | 0.056 |
|  |  | π^*^C_14_-C_15_ | π^*^C_3_-C_10_ | 61.22 | 0.03 | 0.057 |
|  |  |  |  | **𝝨E(2)=177.00** |  |  |
|  |  |  |  |  |  |  |
| Ni^do^Ca^enc^C | LP(1) ^*^$\to$ ẟ^*^ | LP(1)Ni_25_ | ẟ^*^C_6_-Ni_25_ | 99.60 | 1.90 | 0.397 |
|  |  | LP(1)Ni_25_ | ẟ^*^C_17_-Ni_25_ | 94.56 | 1.61 | 0.358 |
|  |  |  |  | **𝝨E(2)=194.16** |  |  |
|  |  |  |  |  |  |  |
|  | ẟ$\to$ ẟ^*^ | ẟC_5_-C_6_ | ẟ^*^C_6_-Ni_25_ | 75.48 | 1.99 | 0.351 |
|  |  |  |  | **𝝨E(2)=75.48** |  |  |
|  |  |  |  |  |  |  |
| Ag^dec^Ni^do^Ca^enc^C | π^*^ $\to$ π^*^ | π^*^C_22_-C_23_ | π^*^C_18_-C_19_ | 60.98 | 0.01 | 0.060 |
|  |  | π^*^C_20_-C_21_ | π^*^C_18_-C_19_ | 61.99 | 0.01 | 0.060 |
|  |  | π^*^C_20_-C_21_ | π^*^C_2_-C_9_ | 32.03 | 0.02 | 0.045 |
|  |  |  |  | **𝝨E(2)=155.00** |  |  |
|  |  |  |  |  |  |  |
| H@C | π^*^ $\to$ π^*^ | π^*^C_23_-C_24_ | π^*^C_21_-C_22_ | 103.87 | 0.01 | 0.066 |
|  |  | π^*^C_23_-C_24_ | π^*^C_4_-C_11_ | 34.97 | 0.01 | 0.039 |
|  |  |  |  | **𝝨E(2)=138.84** |  |  |
|  |  |  |  |  |  |  |
|  | LP^*^(1)$\to$ ẟ^*^ | LP^*^(1)C_13_ | ẟ^*^C_17_-C_18_ | 30.96 | 0.12 | 0.094 |
|  |  |  |  | **𝝨E(2)=30.96** |  |  |
|  |  |  |  |  |  |  |
| H@CaC | π^*^ $\to$ π^*^ | π^*^C_14_-C_15_ | π^*^C_3_-C_10_ | 63.61 | 0.02 | 0.059 |
|  |  | π^*^C_13_-C_18_ | π^*^C_1_-C_8_ | 46.83 | 0.01 | 0.049 |
|  |  |  |  | **𝝨E(2)=110.44** |  |  |
|  |  |  |  |  |  |  |
|  | LP^*^(1)$\to$ ẟ^*^ | LP(1)C_23_ | ẟ^*^C_21_-C_22_ | 42.96 | 0.20 | 0.125 |
|  |  |  |  | **𝝨E(2)=42.96** |  |  |
|  |  |  |  |  |  |  |
| H@NiCaC | ẟ$\to$ LP^*^(6) | ẟC_6_-C_7_ | LP^*^(6)Ni_25_ | 100.40 | 0.85 | 0.387 |
|  |  |  |  | **𝝨E(2)=100.40** |  |  |
|  |  |  |  |  |  |  |
|  | LP(5)$\to$ ẟ^*^ | LP(5)Ni_25_ | ẟ^*^C_6_-C_7_ | 44.00 | 0.14 | 0.104 |
|  |  |  |  | **𝝨E(2)=44.00** |  |  |
|  |  |  |  |  |  |  |
|  | LP(1)$\to$ π^*^ | LP(1)C_11_ | π^*^C_5_-C_6_ | 47.95 | 0.08 | 0.088 |
|  |  |  |  | **𝝨E(2)=47.95** |  |  |
|  |  |  |  |  |  |  |
| H@AgNiCaC | ẟ$\to$ LP(2) | ẟC_17_-N_25_ | LP(2)C_7_ | 58.25 | 0.06 | 0.065 |
|  |  |  |  | **𝝨E(2)=58.25** |  |  |
|  |  |  |  |  |  |  |
|  | LP(1)$\to$ π^*^ | LP(1)C_11_ | π^*^C_5_-C_6_ | 87.18 | 0.09 | 0.089 |
|  |  |  |  | **𝝨E(2)=87.18** |  |  |
|  |  |  |  |  |  |  |
|  | π^*^ $\to$ π^*^ | π^*^C_3_-C_14_ | π^*^C_4_-C_10_ | 75.20 | 0.02 | 0.052 |
|  |  |  |  | **𝝨E(2)=75.20** |  |  |
|  |  |  |  |  |  |  |
| H_2_@C | π^*^ $\to$ π^*^ | π^*^C_1_-C_8_ | π^*^C_21_-C_22_ | 69.93 | 0.01 | 0.044 |
|  |  | π^*^C_19_-C_20_ | π^*^C_21_-C_22_ | 57.61 | 0.01 | 0.040 |
|  |  | π^*^C_14_-C_15_ | π^*^C_3_-C_10_ | 54.23 | 0.02 | 0.047 |
|  |  |  |  | **𝝨E(2)=181.77** |  |  |
|  |  |  |  |  |  |  |
| H_2_@CaC | π $\to$ LP(1) | πC_17_-C_18_ | LP(1)C_13_ | 66.69 | 0.08 | 0.077 |
|  |  |  |  | **𝝨E(2)=66.69** |  |  |
|  |  |  |  |  |  |  |
|  | π^*^ $\to$ π^*^ | π^*^C_20_-C_21_ | π^*^C_1_-C_8_ | 150.61 | 0.01 | 0.049 |
|  |  |  |  | **𝝨E(2)=150.61** |  |  |
|  |  |  |  |  |  |  |
|  | LP(1) $\to$ π^*^ | LP(1)C_16_ | π^*^C_17_-C_18_ | 87.68 | 0.11 | 0.096 |
|  |  |  |  | **𝝨E(2)=87.68** |  |  |
|  |  |  |  |  |  |  |
| H_2_@NiCaC | ẟ$\to$ ẟ^*^ | ẟC_7_-Ni_25_ | ẟ^*^C_17_-Ni_25_ | 118.72 | 0.72 | 0.273 |
|  |  |  |  | **𝝨E(2)=118.72** |  |  |
|  |  |  |  |  |  |  |
|  | π^*^ $\to$ π^*^ | π^*^C_6_-C_18_ | π^*^C_22_-C_23_ | 104.84 | 0.02 | 0.057 |
|  |  | π^*^C_7_-C_19_ | π^*^C_20_-C_21_ | 91.21 | 0.03 | 0.058 |
|  |  |  |  | **𝝨E(2)=196.05** |  |  |
|  |  |  |  |  |  |  |
| H_2_@AgNiCaC | π^*^ $\to$ π^*^ | π^*^C_3_-C_10_ | π^*^C_4_-C_11_ | 74.46 | 0.02 | 0.056 |
|  |  | π^*^C_22_-C_23_ | π^*^C_18_-C_19_ | 103.21 | 0.01 | 0.062 |
|  |  | π^*^C_22_-C_23_ | π^*^C_4_-C_11_ | 60.42 | 0.02 | 0.047 |
|  |  |  |  | **𝝨E(2)=238.09** |  |  |

**Table S2:** Tabulated Charge transfer analysis

| Systems | Occupancy | q_ECT_ (é) |
| --- | --- | --- |
| C | 0.99688 | -0.004 |
| Ca^enc^C | 0.99623 | -0.012 |
| Ni^do^Ca^enc^C | 0.99695 | -0.0045 |
| Ag^dec^Ni^do^Ca^enc^C | 0.99500 | -0.1100 |
| H@C | 0.89900 | 0.1000 |
| H@Ca^enc^C | 0.86005 | 0.0016 |
| H@Ni^dop^Ca^enc^C | 0.11005 | 0.0019 |
| H@Ag^dec^Ni^dop^Ca^enc^C | 0.12004 | 0.0234 |


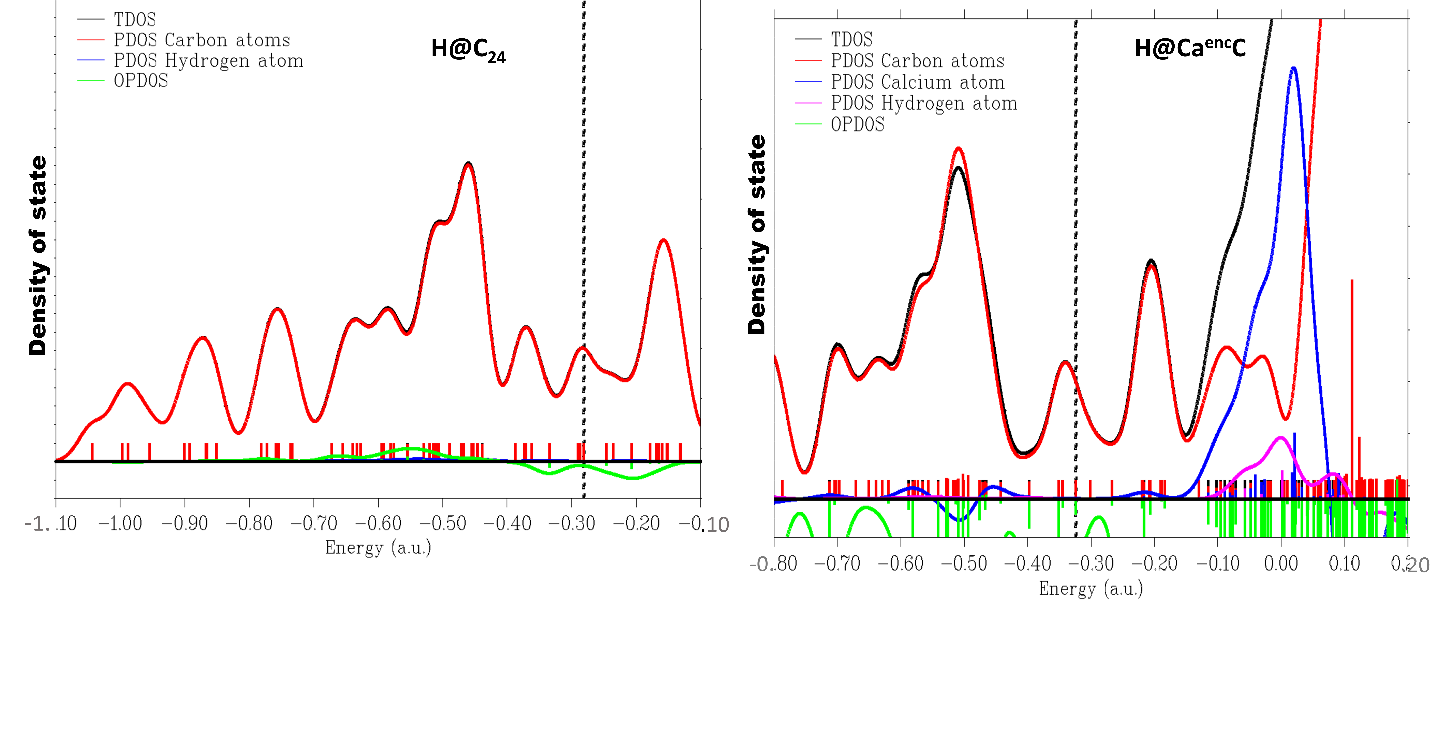

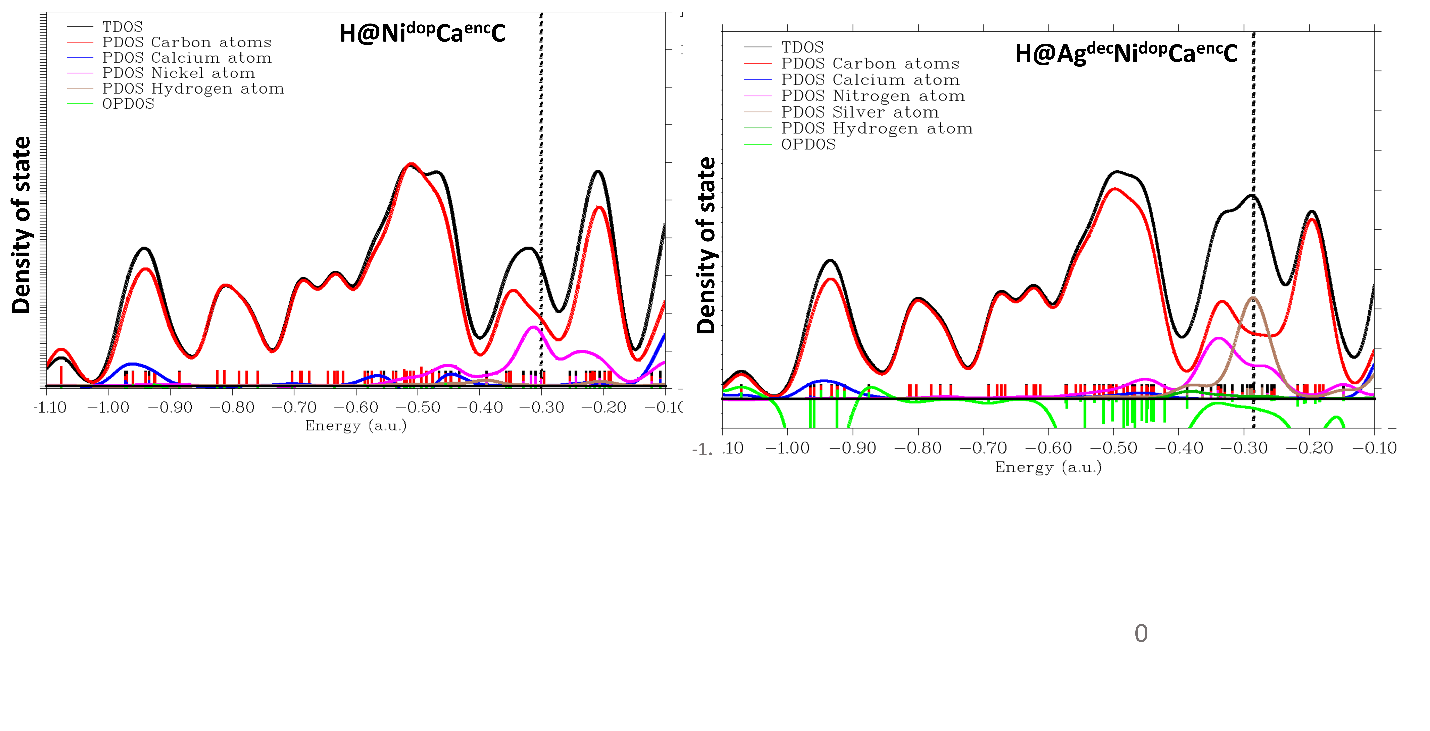


**Figure S1:** Density of state plots for the studied systems showing the representative partial density of state algorithm.
